# Oryzalin-induced polyploidy in *Borago officinalis* reveals cell-wall remodelling via immunofluorescence microscopy

**DOI:** 10.3389/fpls.2025.1676435

**Published:** 2025-11-04

**Authors:** Josef Baltazar Šenkyřík, Anna Milewska-Hendel, Daniel Král, Vladan Ondřej

**Affiliations:** ^1^ Department of Botany, Faculty of Science, Palacký University Olomouc, Olomouc, Czechia; ^2^ Institute of Biology, Biotechnology and Environmental Protection, Faculty of Natural Sciences, University of Silesia in Katowice, Katowice, Poland

**Keywords:** artificial polyploidization, medicinal plants, leaf histo-anatomy, pectin immunolabelling, stomatal traits, flow cytometry, plant cell wall dynamics, *in vitro* mutagenesis

## Abstract

**Introduction:**

Artificial polyploidisation is a powerful biotechnological approach for improving morphological and physiological traits in medicinal plants. We investigated the consequences of chemically induced whole-genome duplication in Borago officinalis L.

**Methods:**

Tetraploidy was induced in vitro using oryzalin. Flow cytometry verified the establishment of mixoploid and stable tetraploid subclones. Selected tetraploids were evaluated for morphology, anatomy, and cellular features using light/confocal microscopy and immunofluorescence labelling of pectic epitopes (homogalacturonan and rhamnogalacturonan I).

**Results:**

Relative to diploids, tetraploids displayed thicker, darker green leaves, increased trichome density, and a distinct growth habit. Microscopy showed significantly enlarged stomata with reduced density, expanded vascular tissues, and altered mesophyll organisation. Immunofluorescence revealed distinct patterns of cell-wall remodelling in tetraploid tissues.

**Discussion:**

These findings illuminate the structural and histochemical consequences of genome-dosage changes in B. officinalis and highlight the potential of chemically induced polyploidy to enhance agronomic and pharmaceutical traits. The work provides a platform for future applications in plant metabolic engineering and molecular pharming.

## 1 Introduction

Polyploidy, the presence of more than two complete sets of chromosomes within an organism, is a key driver of diversification and adaptation in plant biology (Soltis et al., 2009). The deliberate induction of polyploidization, particularly through chemical treatments, has become an established approach in plant breeding, enhancing desirable traits such as growth vigor, stress tolerance, and secondary metabolite production (Comai, 2005; Bharadwaj, 2015; Sattler et al., 2016; Niazian and Nalousi, 2020). *Borago officinalis* L. (borage; Boraginaceae) is an annual medicinal herb valued for its oil-rich in γ-linolenic acid (GLA) with recognized anti-inflammatory benefits; originally Mediterranean, it is now cultivated worldwide for pharmaceutical and nutraceutical use (Tavan et al., 2015).

*In vitro* cultivation and micropropagation enable rapid multiplication of selected genotypes and chemotypes in limited space and time (Rout et al., 2000). Several studies have reported a wide spectrum of *in vitro* methodologies for *B. officinalis*, spanning callus induction and plant regeneration, somatic and zygotic embryogenesis, anther-culture androgenesis yielding haploid and doubled-haploid plants, and *in-vitro* assessments of lipid accumulation (Knipp and Honermeier, 2002; Al-Mohammed Maher et al., 2014; Eshaghi et al., 2015; Abdollahi et al., 2017, 2021; Hoveida et al., 2017; Nasser et al., 2019; Quinn et al., 1989; Whipkey et al., 1988); however, to our knowledge, artificial *in vitro* polyploidization of *B. officinalis* (2n = 2x = 16) has not been specifically addressed (Abdollahi et al., 2017). The induction of polyploidy, a significant tool in plant breeding, can be achieved through mitotic chromosome doubling in somatic tissues or meiotic processes generating 2n gametes (Ramsey and Schemske, 1998; Trojak-Goluch et al., 2021).

Traditionally, colchicine has been used for polyploid induction; however, growing concerns about its toxicity have led to the adoption of alternative agents. Among these, oryzalin (3,5-dinitro-N_4_N_4_-dipropylsulfanilamide), a dinitroaniline herbicide, induces polyploidy by binding to tubulin and disrupting microtubule polymerization, enabling chromosome doubling at significantly lower concentrations than colchicine (Tosca et al., 1995; Švécarová et al., 2018). Its application has been successfully demonstrated across a wide array of plant species. Among medicinal plants, notable examples include *Humulus lupulus* L (Švécarová et al., 2019), *Hypericum* species (Meyer et al., 2009), *Thymus vulgaris* L (Navrátilová et al., 2021), *Ajuga reptans* L (Švécarová et al., 2018), *Dalbergia latifolia* (Riastiwi et al., 2024), and *Cannabis sativa* L (Parsons et al., 2019). While not exhaustive, this selection highlights the versatility of oryzalin in inducing polyploidy across diverse medicinal taxa. Flow cytometry is the most reliable and widely used method for assessing the effectiveness of induced polyploidization (Tosca et al., 1995; Doležel et al., 1998; Dhooghe et al., 2011).

Artificial polyploidization often results in phenotypic modifications, such as increased cell size and changes in organ morphology, a phenomenon known as the “gigas” effect (Levin, 2002). These changes can influence key anatomical structures, including stomata and trichomes, as well as the internal anatomy of leaves and petioles (Tavan et al., 2015; Švécarová et al., 2019; Trojak-Goluch et al., 2021). A mechanistic link between genome duplication and these macroscopic traits resides in altered regulation of primary cell-wall biosynthesis and remodelling: gene dosage can shift the expression of enzymes involved in cell-wall synthesis and modification, thereby altering the assembly, cross-linking, and degree of methyl-esterification of matrix polysaccharides. Such remodelling changes wall mechanics (extensibility, stiffness) and cell–cell adhesion, thereby constraining or enabling tissue growth and patterning (Darley et al., 2001; Cosgrove, 2005, 2016; Wolf et al., 2009; Bidhendi and Geitmann, 2016). In this context, immuno-epitope mapping of pectic domains using antibodies against homogalacturonan (HG) with different degrees of methyl-esterification (JIM5 for low methyl-esterified HG and JIM7 for high methyl-esterified HG) and against rhamnogalacturonan I (RG-I) side chains (LM5 for galactan and LM6 for arabinan), as well as antibodies recognizing arabinogalactan proteins (AGPs; LM2, JIM13), provides sensitive, tissue-level readouts of cell-wall architecture relevant to expansion and vascular differentiation (Knox et al., 1991; Yates et al., 1996; Jones et al., 1997; Willats and Knox, 1999). Understanding these structural changes is crucial, as they may affect not only plant physiology but also the accumulation of bioactive compounds (Madani et al., 2021). Nonetheless, the relationship between polyploidy and anatomical traits is multifaceted, varying across species, cultivars, and environmental conditions (Fernandes et al., 2023).

Building on this mechanistic rationale, our first objective in *B. officinalis* was to establish a rigorous, quantitative baseline linking genome duplication to tissue anatomy and primary cell-wall architecture. Cell-wall organisation regulates cell expansion and tissue mechanics and provides a direct route by which polyploidy can manifest the ‘gigas’ phenotype at the organ scale (Mohnen, 2008; Cosgrove, 2016). Accordingly, we induced tetraploidy with oryzalin, verified ploidy by flow cytometry, and tested the hypothesis that genome duplication remodels wall composition and organisation in a tissue-specific manner. We combined stomatal morphometrics and histology with quantitative immunofluorescence mapping of established epitopes (JIM5, JIM7, LM5, LM6, LM2, JIM13) and statistically compared all lines across defined tissues to detect region-specific shifts in cell-wall architecture (Knox et al., 1991; Yates et al., 1996; Jones et al., 1997; Willats et al., 1998; Willats and Knox, 1999). This staged design establishes a mechanistic baseline and provides the context needed to interpret subsequent changes in gene expression and γ-linolenic acid.

## 2 Material and methods

### 2.1 Plant material

The plant material used in this study was obtained from immature seeds collected from plants that were grown from seeds purchased from SEMO a.s. (Smržice, Czech Republic). As *B. officinalis* is not subdivided into cultivars or breeding lines, no cultivar designation was applicable. Donor plants were vigorous, disease-free, and free from nutrient deficiency symptoms, and were cultivated under standard greenhouse conditions (22–25°C, 16 h photoperiod with natural daylight supplemented by sodium lamps, and regular irrigation). To maximize *in vitro* germination and circumvent dormancy, fruits were collected before complete schizocarp disarticulation, and only physiologically immature nutlets were selected, identified by a green to green-brown color and a pliable, non-hardened testa, while mature, black, hardened nutlets were explicitly avoided. From these immature nutlets, we excised nearly fully formed but still immature zygotic embryos, which were used as explants to initiate *in vitro* cultures. The immature seeds were surface sterilized by immersion in 70% ethanol for 1 minute, followed by a rinse with sterile distilled water. They were then treated with 20% (v/v) solution of commercial bleach (Savo Original, containing approximately 5% sodium hypochlorite, resulting in a final NaOCl concentration of about 1%) and a few drops of Tween-20 on a shaker (240 rpm) for 20 minutes. This was followed by three rinses with sterile distilled water to ensure complete removal of the sterilizing agent. Under a laminar-flow cabinet, nutlets were opened, and zygotic embryos were excised aseptically under a stereomicroscope with sterile instruments. Then, they were immediately transferred to the culture medium.

### 2.2 Culture methods

Embryos were placed individually into De Wit culture tubes (Duchefa, Haarlem, The Netherlands) containing Murashige and Skoog van der Salm Modification (MSvdSM) propagation medium including vitamins (Van Der Salm et al., 1994). This medium differs from standard Murashige and Skoog medium (Murashige and Skoog, 1962) primarily by the use of a chelated iron source (FeEDDHA instead of FeSO^4^/Na_2_EDTA), which provides greater stability and bioavailability of iron. Based on earlier reports and preliminary experience, MSvdSM was chosen as a more suitable medium for *B. officinalis* explants. The medium was enriched with 30 g·L^-^¹ sucrose, 8 g·L^-^¹ agar, 20 mg·L^-^¹ ascorbic acid, 0.01 mg·L^-^¹ indole-3-butyric acid (IBA), 0.01 mg·L^-^¹ 6-benzyladenine (BA), and 0.1% Plant Preservative Mixture (PPM) (Plant Cell Technology, Inc.). Ascorbic acid (ASC) was added to mitigate oxidative stress and phenolic browning during culture initiation, which is in line with previous reports on the use of ASC in plant tissue culture (Pasternak, 2025). The pH of the medium was adjusted to 5.8 before autoclaving. The medium was also augmented with antibiotics (ampicillin, 0.133 g·L^-^¹; chloramphenicol, 0.066 g·L^-^¹) for controlling endogenous bacterial contamination. Antibiotics were dissolved in 0.002 L of dimethyl sulfoxide (DMSO), then diluted with sterile distilled water to a final volume of 30 mL and subsequently sterilised by filtration through a 0.22 µm membrane filter in a laminar flow hood.

Culture tubes were placed in a controlled growth chamber at 22 ± 2°C under a photoperiod of 16 h light and 8 h darkness, with light intensity ranging between 32 and 36 μmol·m^-^²·s^-^¹. Germinated plantlets were subjected to multiplication through repeated trimming of shoots into segments (20–30 mm) and subculturing every six weeks in 100 mL Erlenmeyer flasks containing 30 mL of the same medium. This method yielded approximately 40–60 plants per seed-derived clone. Thirty individual seed-derived lines were established and labelled 1–30.

### 2.3 Oryzalin treatments to induce polyploids

Eight seed-derived genotypes (lines 1, 3, 7, 18, 21, 24, 25, 27) were selected for oryzalin treatment *a priori* based on practical criteria required for induction and downstream replication: confirmed health status, survival during pre-culture, and consistent micropropagation capacity (not on final morphology). For each line, 30 nodal segments were used, and an additional 10 segments were used as controls. Nodal segments with 2–3 nodes were decapitated to remove the apex and used as experimental explants. Oryzalin (Duchefa, Haarlem, The Netherlands) stock solution (10 mM) was prepared according to Greplová et al. (2009), and working concentrations were derived from this stock solution.

Explants were cultivated for two weeks in a hormone-free MSvdSM propagation medium containing 0 μM (control) or 20 μM oryzalin. The concentration of 20 μM was selected as an optimal compromise between survival and polyploidization efficiency, based on our previous *in vitro* experiments with *Astragalus membranaceus*, where higher oryzalin concentrations markedly increased mortality while only slightly improving tetraploid yield (Šenkyřík et al., 2024). Oryzalin was incorporated directly into the culture medium prior to autoclaving. The cultures were maintained in a thermostatically controlled room at 22 ± 2°C under a photoperiod of 16 h light and 8 h darkness, with minimal light exposure (below approximately 5 μmol·m^-^²·s^-^¹) to promote shoot elongation and minimize potential degradation of the compound during the treatment period. After two weeks, explants were transferred to MSvdSM propagation oryzalin-free medium in De Wit culture tubes, with one segment per tube. Each regenerated shoot arising from axillary buds was treated as an individual sub-clone.

### 2.4 Flow cytometric analysis

The ploidy level of sub-clones was assessed using flow cytometry. Nuclei were isolated by finely chopping leaf tissue of the sample and the internal standard, *Pisum sativum* cv. Ctirad (2C = 8.76 pg DNA), with a razor blade in a Petri dish containing 500 μL of LB01 buffer, followed by filtration through a 40 µm nylon mesh as described by Doležel et al. (2007). At least 3,000 nuclei were measured for their relative fluorescence intensity after staining with 4′,6-diamidino-2-phenylindole (DAPI) using a Partec CyFlow ML (Partec GmbH, Münster, Germany) equipped with a 488 nm argon ion laser. Data were analyzed using FloMax Software, Version 2.9. Only polyploid and mixoploid plantlets, along with their controls, were selected for further experiments, while diploid regenerants were excluded.

### 2.5 Micropropagation of polyploid plantlets

Polyploid plantlets were propagated on MSvdSM propagation medium. The medium was augmented with antibiotics as described earlier. Subculturing was conducted approximately every five weeks by dividing the elongated shoots into segments of 4 to 5 cm. Discolored or necrotic leaves were removed during the subculturing process.

### 2.6 *In vitro* rooting of polyploids and transplantation to *ex vitro* conditions

Polyploid shoots were rooted on MSvdSM rooting medium supplemented with 2 mg·L^-^¹ indole-3-acetic acid (IAA), 2 mg·L^-^¹ naphthaleneacetic acid (NAA), 2 mg·L^-^¹ IBA, 20 mg·L^-^¹ ascorbic acid, and 30 g·L^-^¹ sucrose. After approximately two weeks, well-rooted plantlets were carefully removed from the medium, and residual agar was rinsed off the roots. Plantlets were transferred to sterilised, moistened perlite and covered with small plastic domes. Following regular watering and sufficient root growth, plants were transplanted into a substrate and acclimatized in growth chambers under controlled conditions (22 ± 2°C, 16 h light/8 h darkness, 32–36 μmol·m^-^²·s^-^¹ light intensity).

### 2.7 Observation of stomata

Microscopic observations were performed after acclimatization to ex vitro conditions on plants that had successfully transitioned from tissue culture. Stomatal impressions from the abaxial epidermis of fully expanded leaves were obtained using the nail varnish technique, as described by Hamill et al. (1992). Observations and measurements of stomata, including their density, size, and morphology, were performed using a Zeiss Axio Imager microscope equipped with a C-Apochromat 20×/N.A. 0.5 objective and a D512 camera (Zeiss, Göttingen, Germany).

### 2.8 Preparation of samples for microscopic analysis

Leaf segments from the middle part, including the midrib and the leaf blade, and the middle part of the petioles were fixed overnight at 4°C using a 3% glutaraldehyde (GA) and 1% paraformaldehyde (PFA) fixative solution prepared in 0.1 M phosphate-buffered saline (PBS). Next, the samples were rinsed three times with 0.1 M PBS for 10 minutes each and dehydrated in a graded ethanol series (10%, 30%, 50%, 70%, 90%, and 100% v/v). Each step was performed twice for 30 minutes at room temperature (RT). Dehydrated samples were infiltrated with a series of ethanol/LR White resin (Polysciences, Warrington, PA, USA) mixtures to ensure complete resin penetration. The samples were gradually infiltrated with LR White resin:100% ethanol series of 1:2, 1:1, 2:1 (v/v) at 4°C for 24 h each. Following this, the samples were transferred to pure LR White resin for two consecutive 24-hour periods at 4°C. Samples were then embedded in pure resin and polymerized for 24–48 h at 57°C. Leaves were cut into 1.5 μm-thick cross-sections using an EM UC6 ultramicrotome (Leica Microsystems). The sections were collected onto poly-L-lysine-coated glass slides (Polysine^®^, Menzel Thermo Scientific, Jiangsu, China). For histological analysis, sections were stained with 0.05% (w/v) Toluidine blue O (TBO; Sigma-Aldrich, St. Louis, MO, USA) for 10 minutes.

#### 2.8.1 Immunohistochemical analysis

Sections were treated for 30 min at RT with blocking buffer (BB) consisting of 2% fetal calf serum and 2% bovine serum albumin in PBS to block any nonspecific binding sites. Samples were then incubated with primary monoclonal antibodies (Plant Probes, Regensburg, Germany; see **Table 1**), diluted 1:20 in blocking buffer at 4°C overnight. Sections were then washed three times for 10 min with BB and incubated for 1.5h at RT with secondary antibody labelled with Alexa Fluor 488 (Jackson Immuno Research Laboratories, West Grove, PA, USA) diluted 1:100 in BB. Slides were then washed three times for 10 minutes each with BB, then with PBS and sterile distilled water (each step three times for 10 minutes). Dried slides were mounted in Fluoromount (Sigma-Aldrich) antifade medium. Negative controls were performed on all samples by incubation with blocking buffer instead of primary antibodies, and the absence of any signals was found. Observations and photo documentation for immunohistochemical and histochemical analysis were carried out using a Nikon Eclipse Ni-U microscope equipped with a Nikon Digital DS-Fi1-U3 camera with corresponding software (Nikon, Tokyo, Japan) and filters for Alexa Fluor 488 (excitation filter 450–490, barrier filter BA520).

**Table 1:** The list of primary antibodies that were used in the study.

Antibody	Epitope
Pectins	
LM5	(1→4)-β-D-galactan (Jones et al., 1997)
LM6	(1→5)-α-L-arabinan (Willats et al., 1998)
JIM5	partially Me-HG/de-esterified HG (Clausen et al., 2003)
JIM7	partially Me-HG (Clausen et al., 2003)
Arabinogalactan Proteins	
JIM13	AGP glycan, (β)GlcA1→3(α)GalA1→2Rha I (Knox et al., 1991)
LM2	β-linked GlcA (Yates et al., 1996)

### 2.9 Statistical analysis

For stomatal traits, 20 microscopic fields of view per genotype were analyzed. These fields originated from multiple leaves of clonally propagated plants, ensuring representative sampling within each line. The total number of stomata measured per genotype ranged from 233 to 675.

For trichome density, 30 defined regions of interest (ROIs) per genotype were analyzed from the abaxial leaf surface. The dataset comprised five ROIs per leaf from six independent leaves per line (n = 6). Trichome counts were converted to densities (trichomes/mm²) based on image calibration in Fiji (ImageJ distribution, LOCI, University of Wisconsin–Madison, USA).

For histological and immunohistochemical analyses, data were obtained from three leaves per genotype originating from independent clonally propagated plantlets (biological replicates). From each leaf, multiple cross-sections (at least three per antibody and tissue type) were prepared, and in each section, several ROIs or measurement lines were analyzed depending on tissue structure and antibody localization. The number of individual measurements, therefore, varied across tissues; for example, only one measurement per section was taken for the main vascular bundle, whereas up to approximately 100 measurements were collected for epidermal tissues.

All histological dimensions and fluorescence-intensity values were quantified in Fiji. Fluorescence data were normalized by background subtraction to minimize signal variability. The ex vitro diploid control (C2) was excluded from histological and immunohistochemical datasets, as embedded sections were not prepared from this material.

All quantitative datasets (stomatal, trichome, histological, and fluorescence parameters) were analyzed using one-way analysis of variance followed by Tukey’s honestly significant difference (HSD) *post-hoc* test implemented in ASTATSA (Vasavada, 2016). Results are expressed as mean ± standard error (SE). Statistical significance relative to the diploid *in vitro* control (24C) is indicated by asterisks (p ≤ 0.05 “*”; p ≤ 0.01 “**”), while pairwise comparisons between the two tetraploid lines (24/7 and 24/13) for histological and fluorescence datasets are indicated by plus signs (p ≤ 0.05 “+”; p ≤ 0.01 “++”).

## 3 Results

### 3.1 Regeneration and ploidy analysis

**Figure 1:**
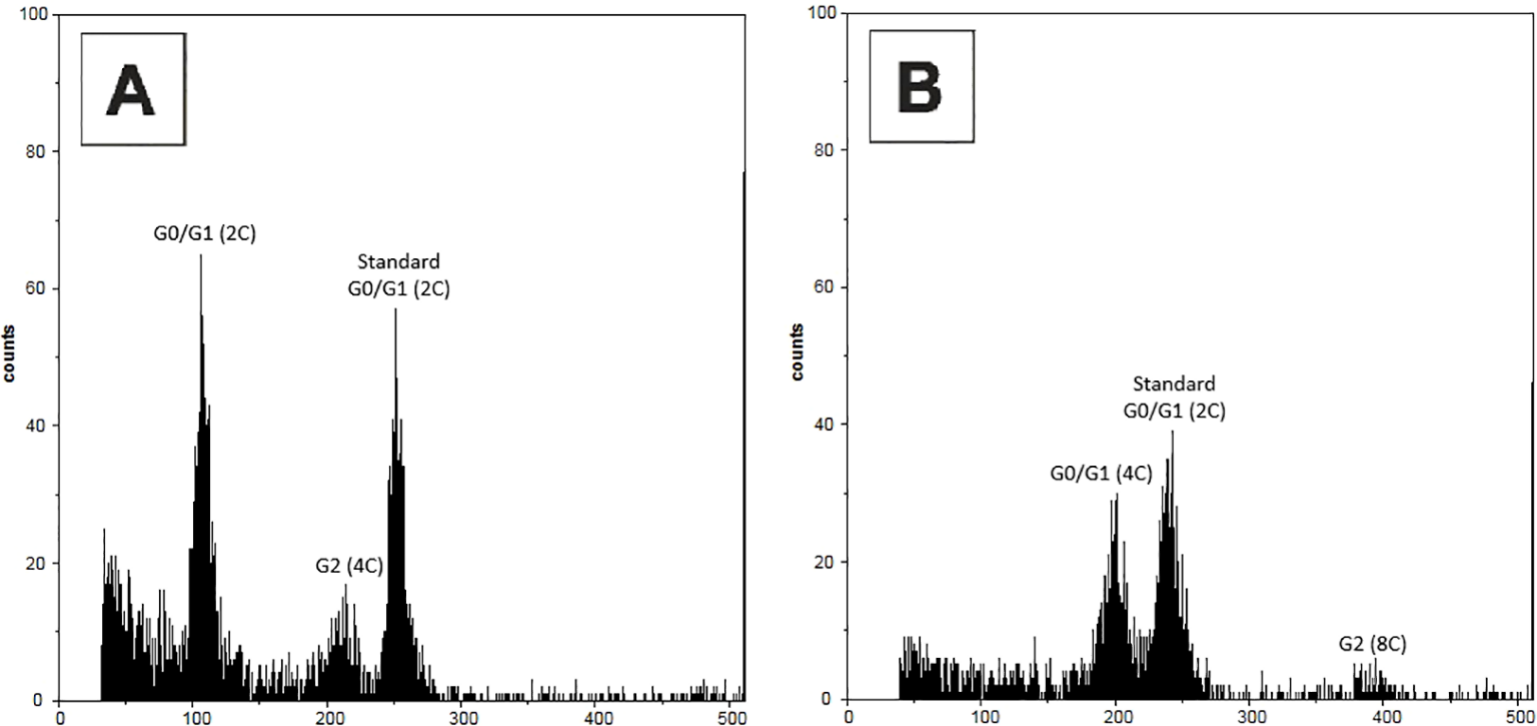
Representative flow cytometry histograms of leaf nuclei of *Borago officinalis* L. grown under *in vitro* conditions, stained with DAPI. **(A)** Diploid control plant not treated with oryzalin, showing peaks corresponding to G0/G1 (2C), G2 (4C), and the internal standard (*Pisum sativum* cv. Ctirad, Standard G0/G1, 2C = 8.76 pg DNA). **(B)** Tetraploid plant, with peaks corresponding to G0/G1 (4C), G2 (8C), and the internal standard (*P. sativum*, Standard G0/G1, 2C).

In total, 108 regenerated sub-clones were obtained from all genotypes subjected to oryzalin treatment. Flow cytometric analysis identified 13 mixoploid sub-clones (12% of all regenerated sub-clones) and six tetraploid sub-clones (6% of all regenerated sub-clones). Detailed results for each genotype are summarized in **Table 2**. Representative histograms of leaf nuclei (**Figure 1**) showed distinct peaks corresponding to the G0/G1 (2C) and G2 (4C) phases in diploid controls (**Figure 1A**), whereas tetraploid sub-clones displayed G0/G1 (4C) and G2 (8C) peaks (**Figure 1B**), with additional higher-order peaks occasionally present due to endopolyploidy, which is common in leaf tissues. Comparison with the internal standard *Pisum sativum* cv. Ctirad (2C = 8.76 pg DNA) allowed a clear distinction between diploid, mixoploid, and tetraploid sub-clones. The coefficients of variation (CV) of the main peaks were within acceptable ranges for reliable analysis: 2.9% (diploid G0/G1, 2C), 2.71% (diploid G2, 4C), 3.6% (tetraploid G0/G1, 4C), and 2.64% (tetraploid G2, 8C).

**Table 2:** Numbers of *B. officinalis* mixoploid and tetraploid sub-clones obtained from *in vitro* cultivation on oryzalin medium for 2 weeks.

Genotype	Viable explants	Viable explants (%)	Viable regenerated sub-clones	Mixoploid sub-clones	Mixoploid sub-clones (%)	Tetraploid sub-clones	Tetraploid sub-clones (%)
1	22	73.33	6	1	16.67	1	16.67
3	14	46.67	2	0	0.00	0	0.00
7	28	93.33	32	5	15.63	1	3.13
18	16	53.33	2	0	0.00	0	0.00
21	20	66.67	23	4	17.39	1	4.35
24	29	96.67	34	3	8.82	3	8.82
25	19	63.33	6	0	0.00	0	0.00
27	15	50.00	3	0	0.00	0	0.00
Sum			108	13	12.04	6	5.56

Sub-clones regenerated from nodal buds of explants exposed to oryzalin were significantly smaller and slower-growing than controls, often swollen, and showed symptoms of vitrification (hyperhydricity). In contrast, all control explants survived the treatment and regenerated normally. Among the analyzed genotypes, genotype 24 produced the highest number of regenerated sub-clones (34), with three determined as mixoploids and three as tetraploids. Genotype 7 followed with 32 regenerated sub-clones, including five mixoploids and one tetraploid. Genotype 21 yielded 23 regenerated sub-clones, of which four were mixoploid and one was tetraploid. Genotype 1 had six regenerated sub-clones, with one identified as mixoploid and one as tetraploid. Other genotypes (3, 18, 25, and 27) did not produce any confirmed tetraploid subclone.

### 3.2 Transfer to *ex vitro* conditions

Out of the regenerated sub-clones, only genotypes from clones 24 and 7 were successfully transferred to ex vitro conditions. These included two control lines: 24C (control clones of genotype 24) and 7C (control clones of genotype 7), as well as three tetraploid sub-clones identified by flow cytometric measurements: 24/5 (the fifth subclone measured), 24/7 (the seventh subclone measured), and 24/13 (the thirteenth subclone measured). Rooting of these lines was achieved on MSvdSM medium supplemented with auxins (IAA, NAA, and IBA at equal concentrations of 2 mg·L^-^¹ each) and ascorbic acid, followed by gradual acclimatization in sterilised perlite and subsequent transplantation into a substrate under controlled growth chamber conditions.

The success rate of transfer to ex vitro conditions was high for control lines 24C and 7C, with all plantlets surviving the acclimatization process. However, the tetraploid sub-clones exhibited a slightly reduced success rate, particularly in 24/13, where approximately 80% of plantlets survived. These differences might reflect the intrinsic variability in physiological responses associated with ploidy levels. Overall, the established ex vitro plants showed robust growth and were phenotypically stable, demonstrating that the applied protocol is effective for the acclimatization of both control and polyploid lines.

### 3.3 Morphological variability in tetraploid lines

**Figure 2:**
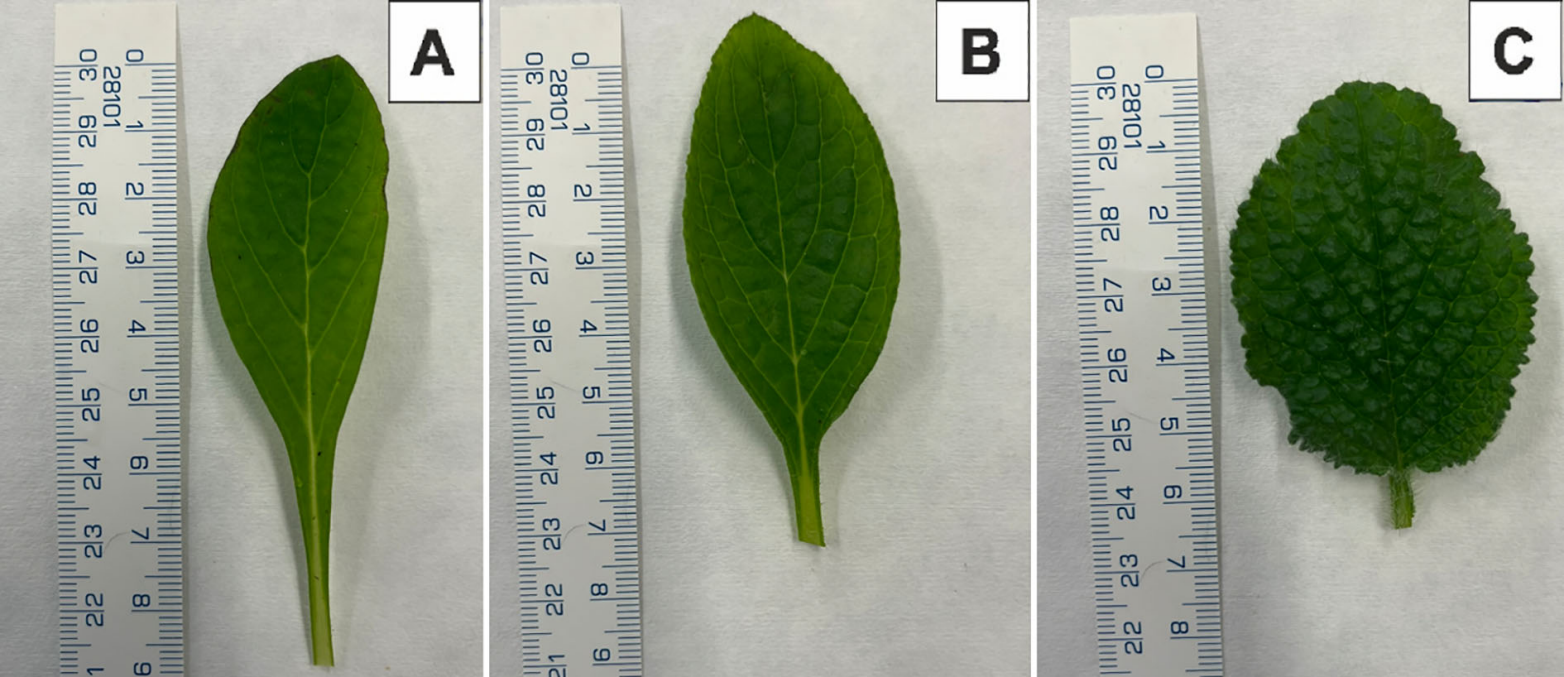
Young leaf morphology of *Borago officinalis* L. plants from ex vitro conditions. **(A)** Control plant 24C. **(B)** Tetraploid sub-clonal line 24/7. **(C)** Tetraploid sub-clonal line 24/13.

**Figure 3:**
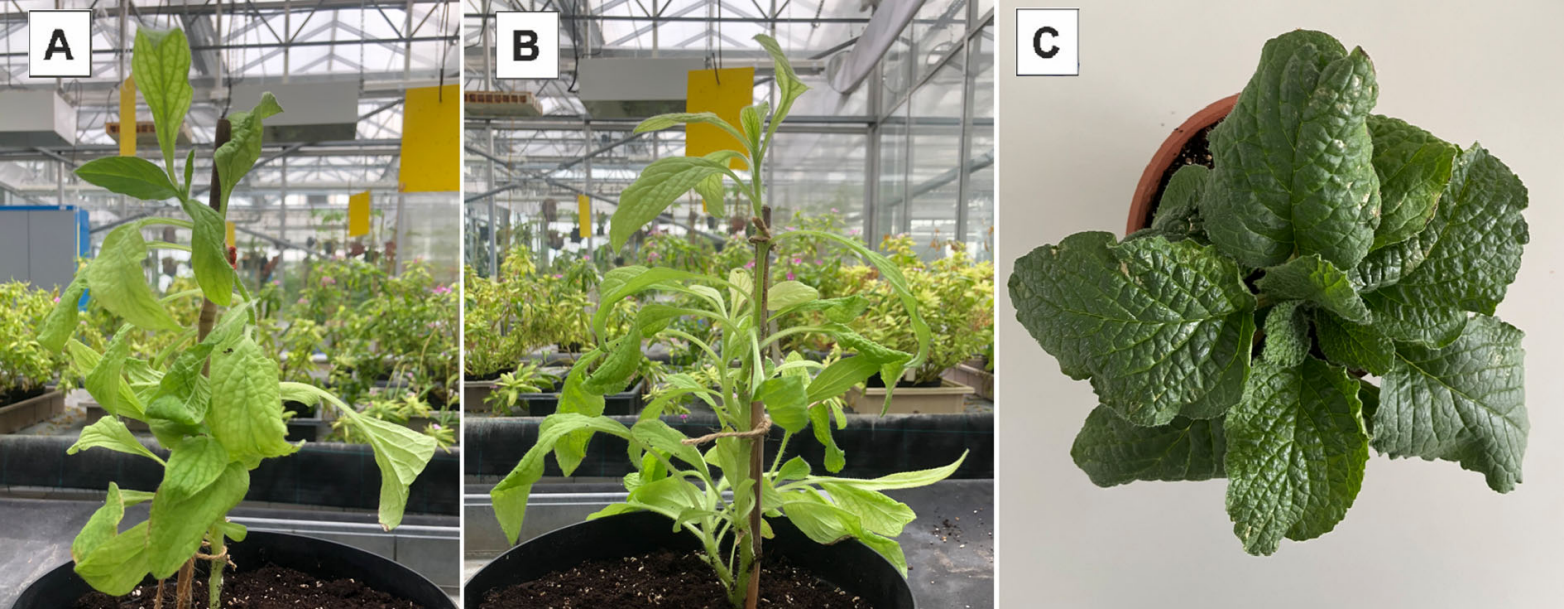
Plants of *Borago officinalis* L. in ex vitro conditions. **(A)** Control plant 24C. **(B)** Tetraploid sub-clonal line 24/7. **(C)** Tetraploid sub-clonal line 24/13.

Representative ex vitro morphology is shown in **Figures 2**, **3**. Leaf shape differed among lines: 24C and 24/7 exhibited lanceolate blades, whereas 24/13 displayed broadly ovate blades with pronounced marginal serration (**Figure 2**). Leaves of the tetraploid lines appeared more pubescent than those of the diploid control (**Figure 2**); see Section 3.5 for the trichome assessment.

In whole plants, 24C and 24/7 showed an erect, branching habit, whereas 24/13 presented a rosette-like architecture with closely spaced leaves (**Figure 3**).

### 3.4 Stomatal characteristics

**Figure 4:**
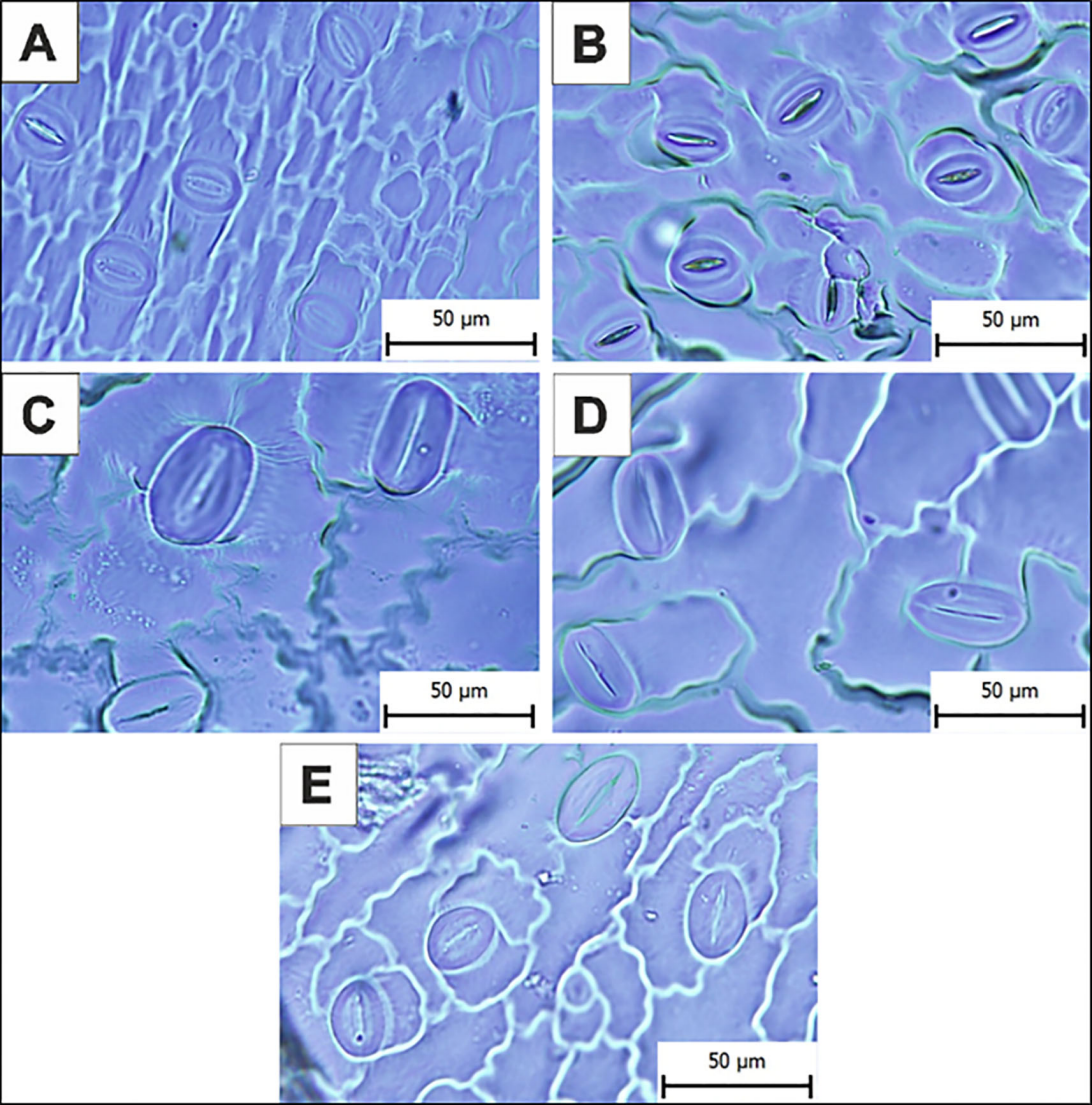
Microphotographs of the stomata on the abaxial epidermis of control and tetraploid sub-clonal lines. **(A)** *In vitro* control plants (C1). **(B)** Outdoor control plants (C2). **(C)** Tetraploid sub-clonal line 24/5. **(D)** Tetraploid sub-clonal line 24/7. **(E)** Tetraploid sub-clonal line 24/13. Scale bar = 50 µm.

Stomatal measurements demonstrated significant differences between control and tetraploid lines (**Table 3**). The average stomatal size in the *in vitro* control plants (C1) was 23.57 ± 2.62 µm in length and 15.24 ± 1.67 µm in width. In the outdoor control plants (C2), stomatal size was similar in length (23.54 ± 3.12 µm) but significantly smaller in width (13.96 ± 2.15 µm, p ≤ 0.01). Tetraploid sub-clonal lines exhibited significantly larger stomata compared to the *in vitro* control (C1), with average lengths of 35.73 ± 5.66 µm (24/5), 34.67 ± 5.00 µm (24/7), and 31.43 ± 4.94 µm (24/13). Corresponding stomatal widths were also significantly larger at 22.54 ± 4.51 µm, 20.97 ± 2.45 µm, and 22.83 ± 2.97 µm, respectively (p ≤ 0.01) (**Figure 4**).

**Table 3:** Stomatal size and density in control and tetraploid sub-clonal lines.

Genotype	Length [µm] (Mean ± SE)	Width [µm] (Mean ± SE)	Density [mm²]
C1	23.57 ± 0.12	15.24 ± 0.08	155.86
C2	23.54 ± 0.12	13.96 ± 0.08**	231.74**
24/5	35.73 ± 0.36**	22.54 ± 0.29**	83.42**
24/7	34.67 ± 0.33**	20.97 ± 0.16**	79.99**
24/13	31.43 ± 0.27**	22.83 ± 0.16**	118.10**

Mean stomatal length (µm), width (µm), and density (stomata/mm²) are presented for outdoor control plants (C2), *in vitro* control plants (C1), and tetraploid sub-clonal lines (24/5, 24/7, 24/13). Values marked with ** indicate a statistically significant difference compared to the *in vitro* control (C1) at p ≤ 0.01.

Stomatal density was highest in the outdoor control plants (C2), at 231.74 stomata/mm², compared to 155.86 stomata/mm² in the *in vitro* control (C1). Tetraploid sub-clonal lines showed a significantly reduced stomatal density, with values of 83.42/mm² (24/5), 79.99/mm² (24/7), and 118.1/mm² (24/13), indicating a trade-off between increased stomatal size and decreased density.

### 3.5 Trichome characteristics

Quantitative evaluation of abaxial trichome density revealed marked differences between the control and tetraploid lines (**Table 4**). The abaxial leaf surface of the *in vitro* control plants (24C) exhibited a relatively sparse indumentum, with a mean trichome density of 2.00 ± 0.09 trichome/mm². In contrast, the tetraploid sub-clonal lines displayed a substantially higher density of trichomes. Line 24/7 reached an average of 14.44 ± 0.51 trichome/mm², whereas line 24/13 attained the highest value of 19.55 ± 0.63 trichome/mm² (p ≤ 0.01). The increase in trichome number was visually apparent under stereomicroscopy, with tetraploid leaves exhibiting a denser and more uniform indumentum, particularly along the interveinal regions of the abaxial surface.

**Table 4:** Trichome density in control diploid (24C) and tetraploid sub-clonal lines (24/7, 24/13).

Genotype	Density [trichomes/mm²] (Mean ± SE)
24C	2.00 ± 0.09
24/7	14.44 ± 0.51**
24/13	19.55 ± 0.63**

Mean trichome density (trichomes/mm² ± SE). Values marked with ** indicate a statistically significant difference compared to the 24C at p ≤ 0.01.

These results indicate that genome duplication strongly enhances trichome initiation and development in B. officinalis, contributing to the characteristic rougher leaf texture observed in the tetraploid lines.

### 3.6 Characteristics of the histological structure

**Figure 5:**
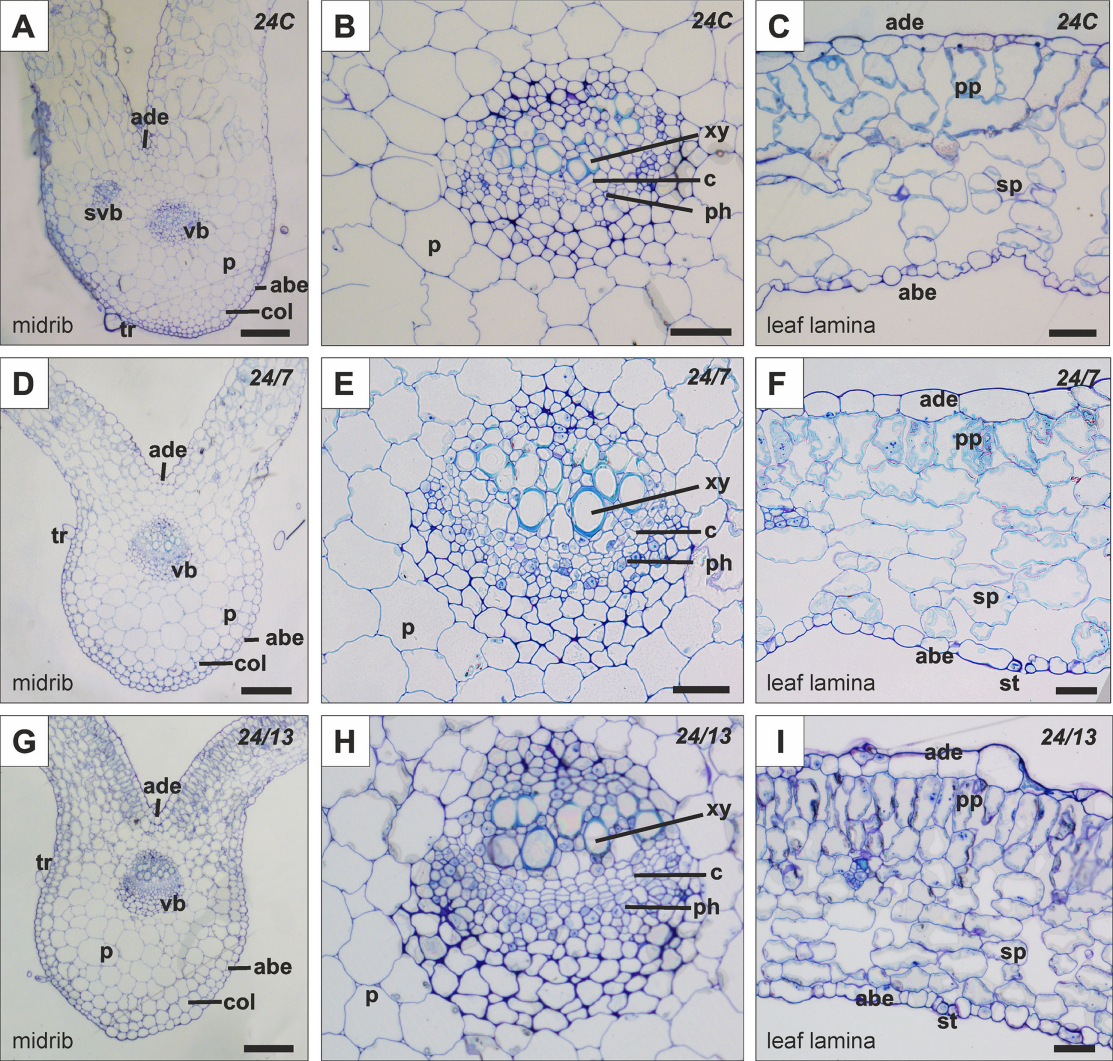
Histology of the leaf lamina and midrib in control 24C **(A–C)** and tetraploid lines 24/7 **(D–F)** and 24/13 **(G–I)**; abe – abaxial epidermis, ade – adaxial epidermis, c – cambium, col – collenchyma, p – parenchyma, ph – phloem, pp – palisade parenchyma, sp – spongy parenchyma, st -stomata, svb – secondary vascular bundle, tr – trichome, vb – vascular bundle, xy – xylem. Scale bars **(A, D, G)** – 200 µm; **(B, C, E, F, H, I)** – 50 µm.

**Figure 6:**
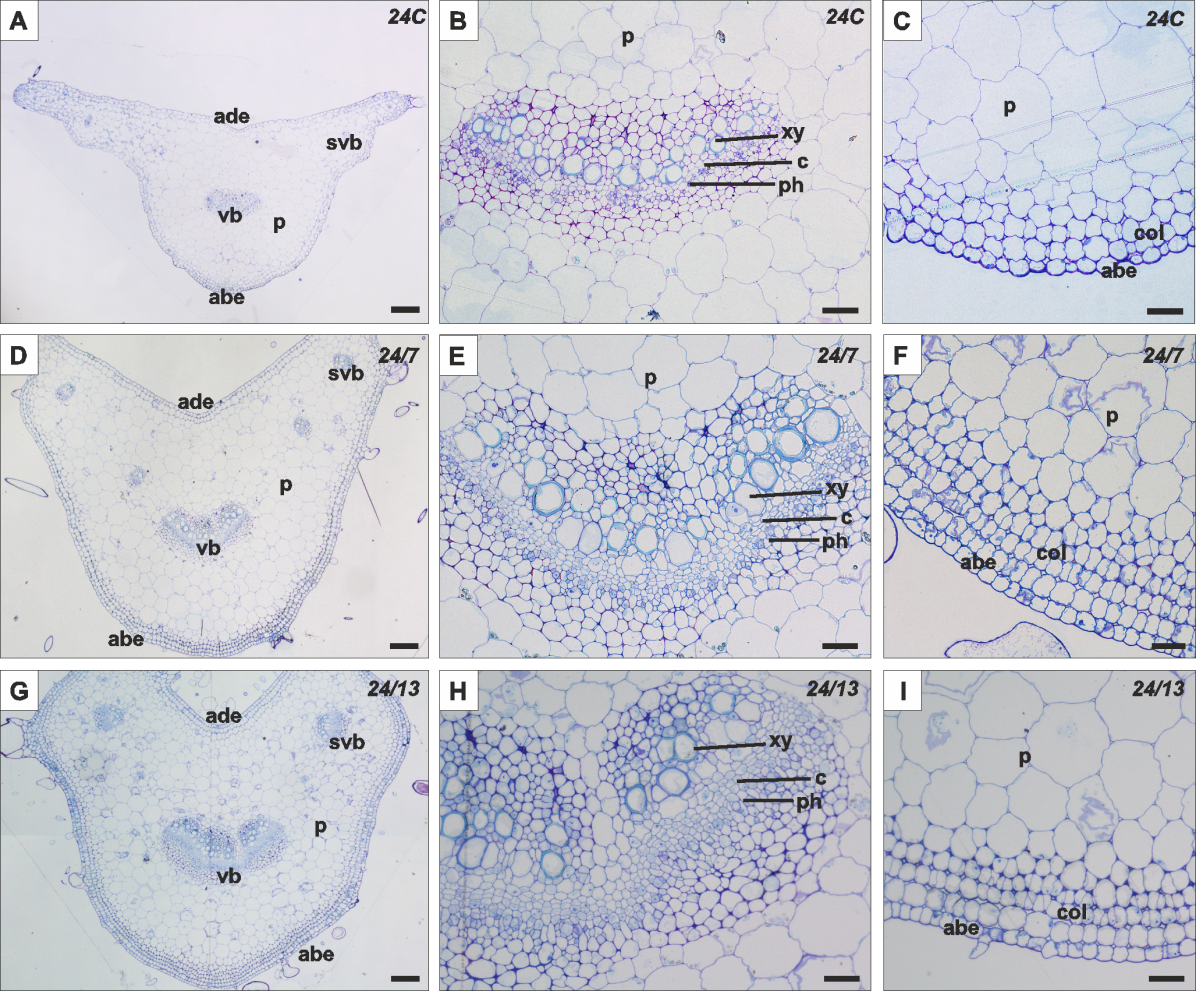
Histology of the petiole in control 24C **(A–C)** and tetraploid lines 24/7 **(D–F)** and 24/13 **(G–I)**; abe – abaxial epidermis, ade – adaxial epidermis, c – cambium, col – collenchyma, p – parenchyma, ph – phloem, pp – palisade parenchyma, sp – spongy parenchyma, st -stomata, svb – secondary vascular bundle, vb – vascular bundle, xy – xylem. Scale bars **(A, D, G)** – 200 µm; **(B, C, E, F, H, I)** – 50 µm.

**Figure 7:**
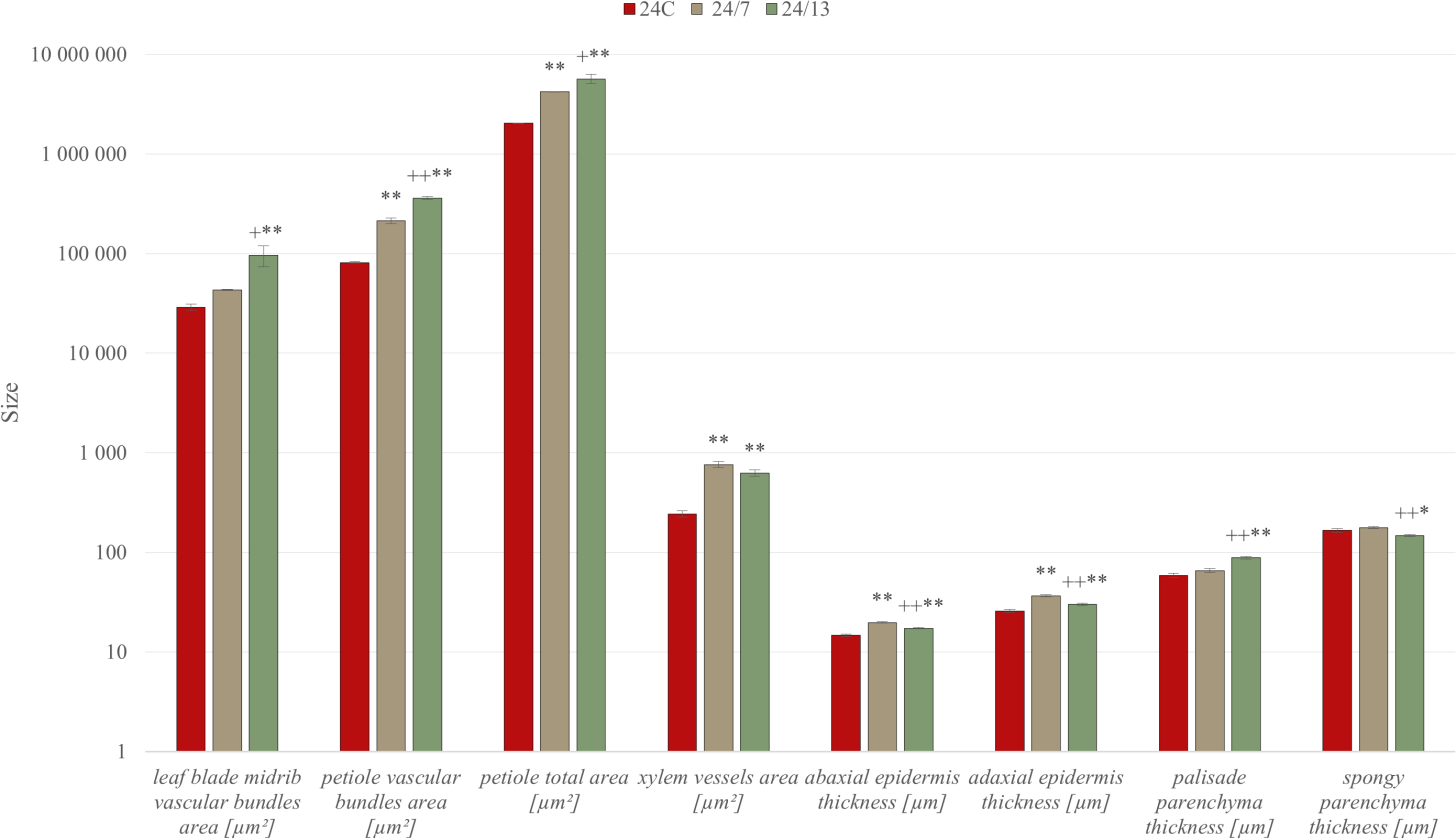
Quantitative anatomical characteristics of diploid and tetraploid *Borago officinalis* leaves and petioles. Histological measurements were performed on transverse sections of *in vitro*-grown plants to assess the areas of vascular bundles in the leaf midrib and petiole, total petiole area, xylem vessel area, and the thickness of abaxial and adaxial epidermis, palisade, and spongy parenchyma. Data are presented as mean ± SE. Significant differences are indicated as (p ≤ 0.05 “*”; p ≤ 0.01 “**”) relative to the diploid control (24C), and (p ≤ 0.05 “+”; p ≤ 0.01 “++”) for comparisons between tetraploid lines (24/7 *vs* 24/13).

Representative histological sections are shown in **Figures 5** (lamina and midrib) and **Figures 6** (petiole), while the corresponding quantitative morphometrics are summarized in **Figure 7**. The B. officinalis leaf has a typical structure for dicotyledonous plants (**Figures 5**). The epidermis contains trichomes, including glandular ones. Below the epidermis, within the midrib, there is collenchyma with unevenly thickened walls that provide elasticity (**Figures 5A, D, G**). The main vascular bundle in the midrib is an open collateral bundle (**Figures 5B, E, H**). The xylem is located on the adaxial side, and the phloem on the abaxial side, with cambium cells between them (**Figures 5B, E, H**). Within the leaf blades, palisade parenchyma and spongy parenchyma are distinguished (**Figures 5D, F, I**).

Histological analysis of the leaf blade and the midrib revealed differences between control (**Figures 5A-C**) and tetraploid lines 24/7 (**Figures 5D–F**) and 24/13 (**Figures 5G–I**). Midrib main vascular bundle area increased in 24/13 relative to 24C (**Figure 6**; p ≤ 0.01), 24/13 exceeded 24/7 (p ≤ 0.05), whereas 24/7 did not differ from 24C (**Figure 6**). Representative transverse sections are shown in **Figures 5B, E, H**. Xylem vessel area was higher in both tetraploid lines than in 24C (**Figure 6**; p ≤ 0.01), with no difference between the tetraploids (**Figure 6**). Qualitatively, both tetraploid lines showed a more conspicuous cambial zone, particularly 24/13, while overall tissue organisation remained comparable to 24C (**Figures 5B, E, H**). Epidermal thickness increased on both surfaces in tetraploids relative to 24C. On the abaxial side, both 24/7 and 24/13 exceeded 24C (**Figure 6**; p ≤ 0.01) and 24/7 > 24/13 (**Figure 6**; p ≤ 0.01). On the adaxial side, both tetraploids again exceeded 24C (**Figure 6**; p ≤ 0.01) and 24/7 > 24/13 (**Figure 6**; p ≤ 0.01). Palisade parenchyma (**Figures 5C, F, I**) was thicker in 24/13 than in 24C (**Figure 6**; p ≤ 0.01), and 24/13 also exceeded 24/7 (**Figure 6**; p ≤ 0.01); 24/7 did not differ from 24C (**Figure 6**). For the spongy parenchyma (**Figure 5C, F, I**), 24C exceeded 24/13 (**Figure 6**; p ≤ 0.05) and 24/7 exceeded 24/13 (**Figure 6**; p ≤ 0.01), while 24/7 did not differ from 24C (**Figure 6**). In representative sections, line 24/13 showed a more compact arrangement of spongy parenchyma cells (**Figure 5I**), consistent with its lower spongy parenchyma thickness relative to 24C (**Figure 6**; p ≤ 0.05).

The histology of the *B. officinalis* petiole is characterized by several distinct layers of tissue (**Figure 7**). The outermost layer is the epidermis, composed of tightly packed cells, and is covered with glandular and non-glandular trichomes. Beneath the epidermis are layers of collenchyma, beneath which lies the cortex, composed of parenchyma cells. In the center of the petiole is a vascular bundle, consisting of xylem on the adaxial side and phloem on the abaxial side, with a layer of cambium in between. In addition to the central vascular bundle, smaller secondary vascular bundles are located along the sides of the petiole.

Morphometric analysis, it was found that the leaf petioles in tetraploid lines 24/7 and 24/13 were significantly larger in total cross-sectional area compared to the control 24C (**Figure 6**; p ≤ 0.01), and 24/13 also exceeded 24/7 (**Figure 6**; p ≤ 0.05). In addition, the petioles in the tetraploid lines varied in the shape of the cross-section, displaying a more developed cross-sectional architecture than 24C (**Figures 7A, D, G**). Different anatomical features were observed within the central vascular bundle, which in lines 24/7 and 24/13 was characterized by a more developed architecture than in the control 24C (**Figures 7B, E, H**). The central vascular bundle area was likewise increased in 24/7 and 24/13 relative to 24C (**Figure 6**; both p ≤ 0.01), with 24/13 exceeding 24/7 (**Figure 6**; p ≤ 0.01); representative transverse sections are shown in **Figures 7B, E, H**. In transverse sections, vessels appeared wider in the tetraploids (**Figures 7B, E, H**), consistent with the larger xylem vessel area quantified in **Figure 6** (24/7 and 24/13 > 24C, p ≤ 0.01; no difference between the tetraploids). In cross-section, the central bundle of tetraploid petioles appeared more developed; vessel elements tended to be wider and the cambial zone more conspicuous, particularly in 24/13 (**Figures 7E, H**). On the abaxial side, tetraploid petioles typically presented four layers of collenchyma rather than the three irregular layers seen in 24C, and collenchyma cells appeared more homogeneous and regularly shaped in 24/7 and 24/13 (**Figures 7C, F, I**).

### 3.7 Immunohistochemical analysis of the cell wall

The study analyzed the distribution of cell wall components in the leaf blade, the midrib and the petiole of the control 24C and tetraploid lines 24/7 and 24/13 leaves. Immunolocalization was performed using specific antibodies enabling the identification of pectins (JIM5, JIM7, LM5, LM6) and arabinogalactan proteins (AGPs) (LM2, JIM13).

**Figure 8:**
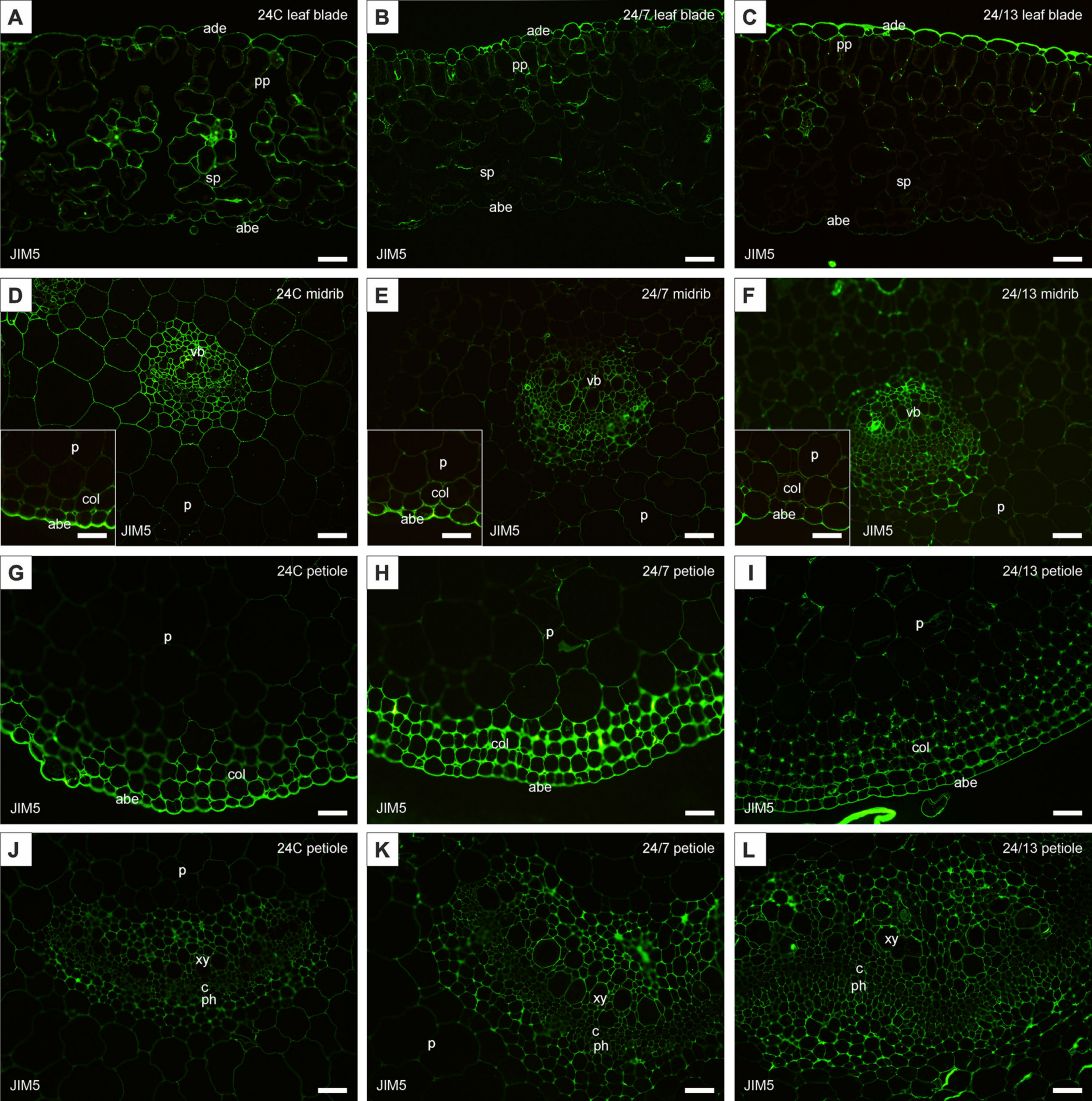
Immunohistochemical localization of JIM5 epitope in control 24C **(A, D, G, J)** and tetraploid lines: 24/7 **(B, E, H, K)** and 24/13 **(C, F, I, L)** in the leaf lamina **(A–C)**, midrib **(D–F)** and petiole **(G–L)**; abe – abaxial epidermis, ade – adaxial epidermis, c – cambium, col – collenchyma, p – parenchyma, ph – phloem, pp – palisade parenchyma, sp – spongy parenchyma, vb – vascular bundle, xy – xylem. Scale bars **(A–L)**, insets **(D–F)** – 50 µm.

**Figure 9:**
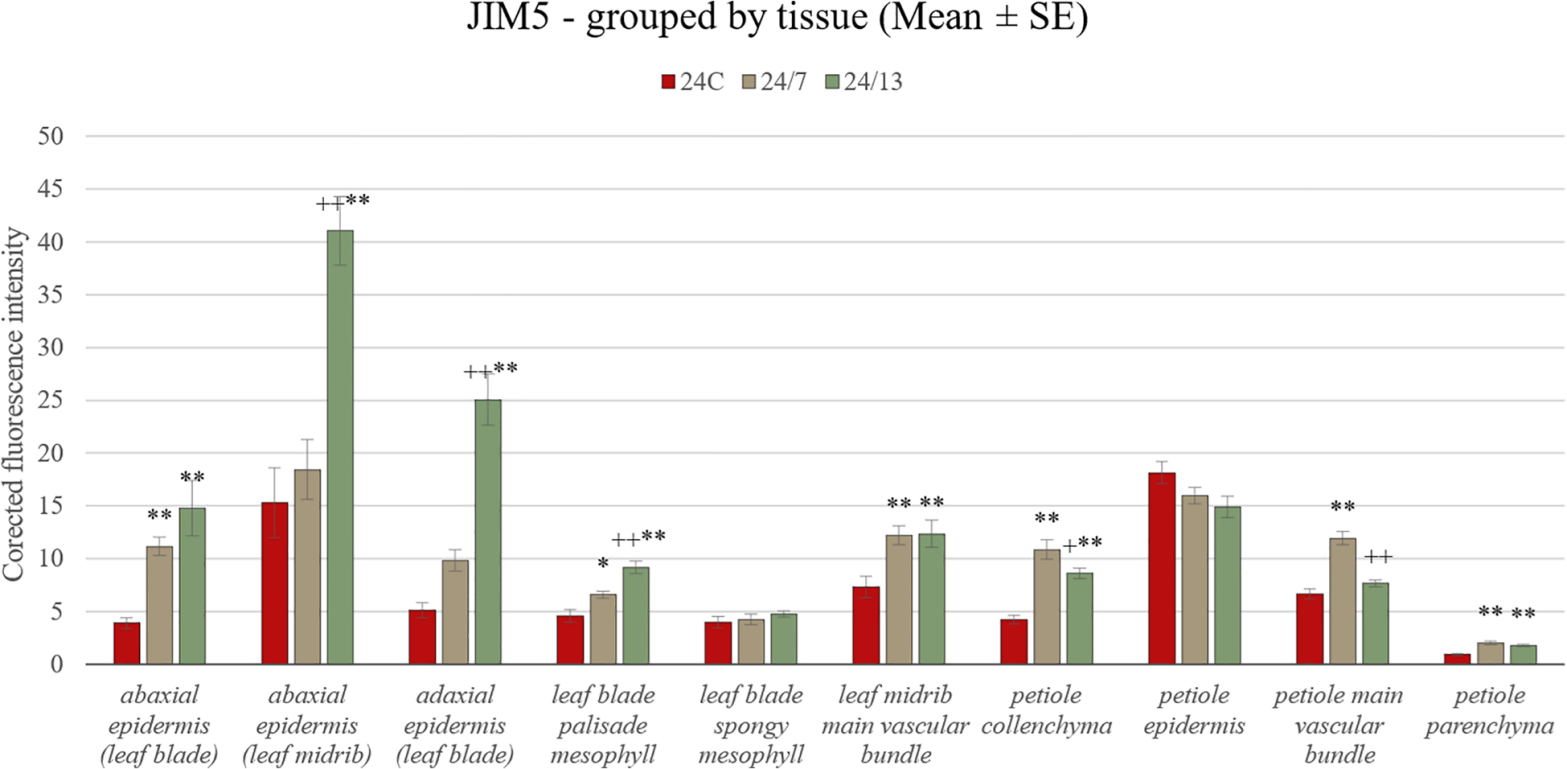
Quantification of JIM5 fluorescence intensity in leaf and petiole tissues of diploid and tetraploid *Borago officinalis*. Corrected mean ± SE fluorescence intensities were determined for the following tissues: abaxial epidermis (leaf blade), abaxial epidermis (leaf midrib), adaxial epidermis (leaf blade), leaf blade palisade mesophyll, leaf blade spongy mesophyll, leaf midrib main vascular bundle, petiole collenchyma, petiole epidermis, petiole main vascular bundle, and petiole parenchyma. The JIM5 antibody recognizes low-methyl-esterified homogalacturonan. Significant differences are indicated as (p ≤ 0.05 “*”; p ≤ 0.01 “**”) relative to the diploid control (24C), and (p ≤ 0.05 “+”; p ≤ 0.01 “++”) for comparisons between tetraploid lines (24/7 *vs* 24/13).

The JIM5 antibody, recognizing low-methyl-esterified homogalacturonan, was detected in both adaxial and abaxial epidermis of the leaf blade as well as in the midrib epidermis of the control and tetraploid leaves (**Figures 8A–C**). Quantitative fluorescence analysis confirmed significant differences between lines (**Figure 9**). In the abaxial epidermis of the blade, both tetraploids displayed markedly higher signals than 24C (mean ± SE: 24C 3.9 ± 0.5, 24/7 11.2 ± 0.9, 24/13 14.8 ± 2.6; p < 0.01), whereas the two tetraploid lines did not differ significantly. In the adaxial epidermis, fluorescence intensities followed a line-dependent gradient (24C 5.1 ± 0.7, 24/7 9.8 ± 1.0, 24/13 25.1 ± 2.4), with 24/13 exhibiting significantly stronger labelling than both 24C and 24/7 (p < 0.01). In the abaxial epidermis of the midrib, 24/13 showed the strongest signal (41.1 ± 3.3), significantly exceeding both 24C (15.3 ± 3.3) and 24/7 (18.4 ± 2.8; p < 0.01), while 24/7 did not differ from 24C (**Figure 8D–F**).

In the palisade mesophyll, a clear line-dependent increase in fluorescence was recorded (24C 4.5 ± 0.5, 24/7 6.9 ± 0.7, 24/13 10.3 ± 0.8; p < 0.01), with both tetraploid lines significantly exceeding the control, and 24/13 surpassing 24/7 (p < 0.01). In contrast, the spongy mesophyll did not show statistically significant differences between lines (p = 0.468), although all genotypes displayed detectable labelling (**Figures 8A–C**). In the main vascular bundle of the midrib, JIM5 labelling differed significantly (p < 0.01), with both tetraploids exhibiting stronger fluorescence than 24C, but no difference between 24/7 and 24/13.

In the petiole, the JIM5 epitope was present across all tissues examined (**Figures 8G–L**). Quantitative analysis revealed no significant differences in the epidermis (p = 0.182). In collenchyma, however, 24/7 displayed the highest fluorescence, which was significantly stronger than both 24C and 24/13 (p < 0.01), while 24/13 showed weaker fluorescence than 24/7 (p < 0.01). Petiole parenchyma displayed a significant line-dependent increase (24C 0.94 ± 0.1, 24/7 1.86 ± 0.2, 24/13 2.06 ± 0.3; p < 0.01), with both tetraploid lines exceeding the diploid control. In the main vascular bundle of the petiole, significant differences were detected (p < 0.01), with 24/13 showing reduced fluorescence compared with 24/7 (p < 0.01).

**Figure 10:**
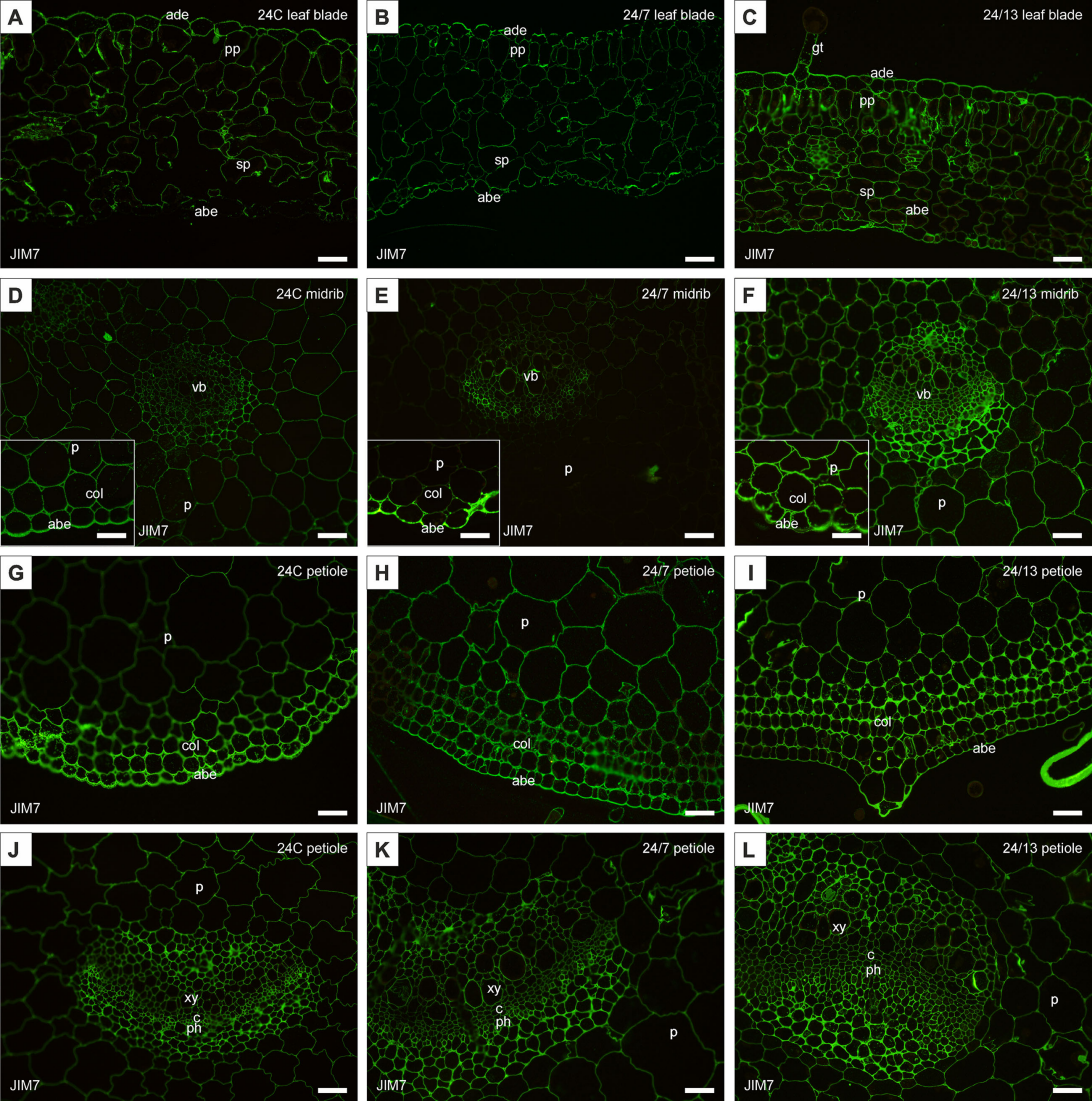
Immunohistochemical localization of JIM7 epitope in control 24C **(A, D, G, J)** and tetraploid lines: 24/7 **(B, E, H, K)** and 24/13 **(C, F, I, L)** in the leaf lamina **(A–C)**, midrib **(D–F)** and petiole **(G–L)**; abe – abaxial epidermis, ade – adaxial epidermis, c – cambium, col – collenchyma, gt – glandular trichome, p – parenchyma, ph – phloem, pp – palisade parenchyma, sp – spongy parenchyma, vb – vascular bundle, xy – xylem. Scale bars **(A–L)**, insets **(D–F)** – 50 µm.

**Figure 11:**
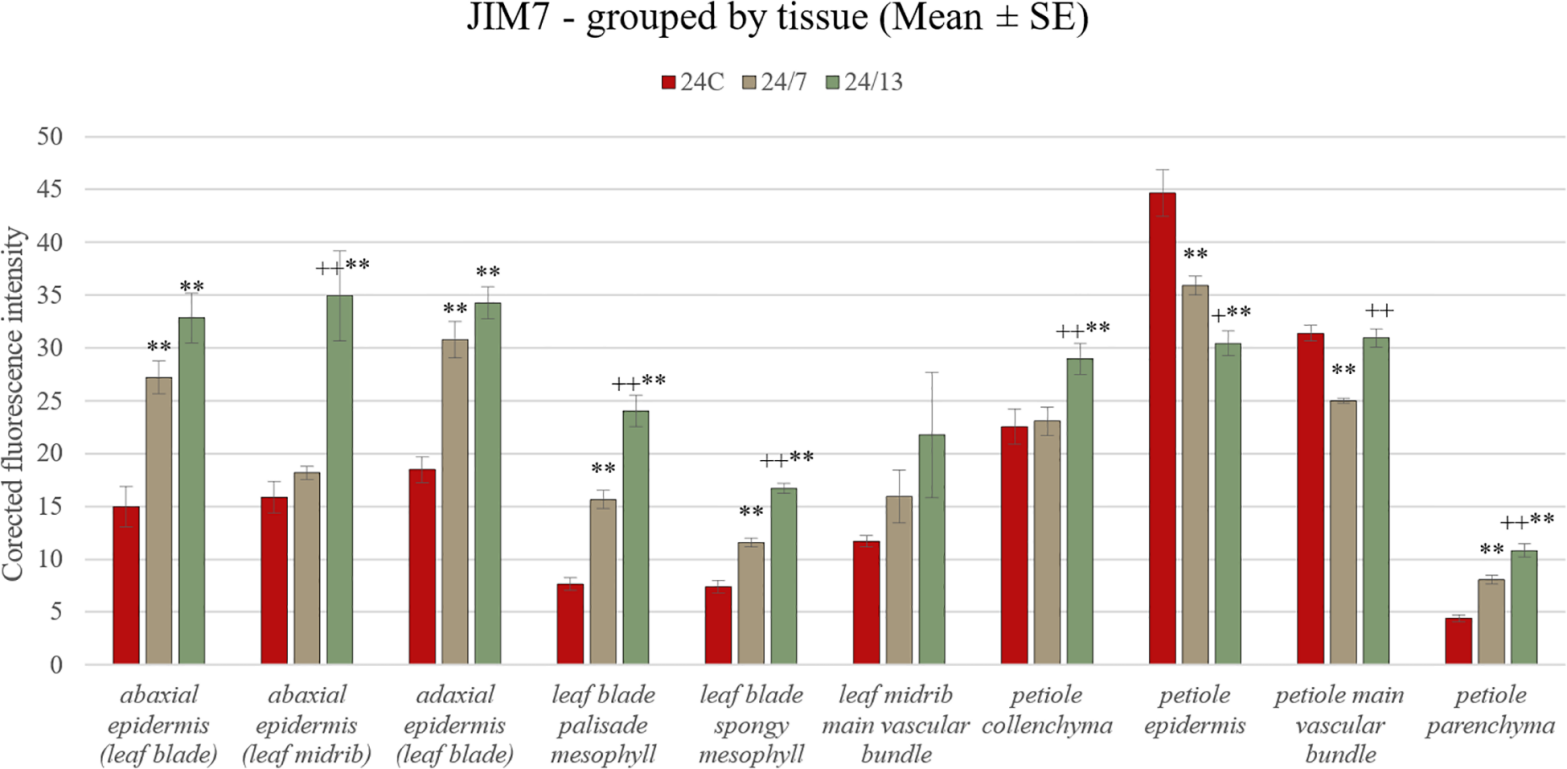
Quantification of JIM7 fluorescence intensity in leaf and petiole tissues of diploid and tetraploid *Borago officinalis*. Corrected mean ± SE fluorescence intensities were measured for abaxial epidermis (leaf blade), abaxial epidermis (leaf midrib), adaxial epidermis (leaf blade), leaf blade palisade mesophyll, leaf blade spongy mesophyll, leaf midrib main vascular bundle, petiole collenchyma, petiole epidermis, petiole main vascular bundle, and petiole parenchyma. The JIM7 antibody recognizes partially methyl-esterified homogalacturonan. Significant differences are indicated as (p ≤ 0.01 “**”) relative to the diploid control (24C), and (p ≤ 0.05 “+”; p ≤ 0.01 “++”) for comparisons between tetraploid lines (24/7 *vs* 24/13).

The JIM7 antibody was strongly detected in the cell walls of the adaxial epidermis and mesophyll layers of all genotypes (**Figures 10A–C**). Quantitative fluorescence confirmed significant increases in both epidermal and mesophyll tissues in tetraploids compared with the diploid control (**Figure 11**). In the abaxial epidermis of the blade, fluorescence intensities were 24C 18.4 ± 1.2, 24/7 28.9 ± 1.6 and 24/13 36.9 ± 1.7 (p < 0.01), with both tetraploids exceeding 24C and 24/13 also significantly higher than 24/7 (p < 0.01). In the adaxial epidermis, a similar line-dependent gradient was observed (24C 18.1 ± 1.3, 24/7 31.0 ± 1.6, 24/13 35.2 ± 1.4; p < 0.01). In the abaxial epidermis of the midrib, 24/13 again exhibited the strongest signal, significantly higher than both 24C and 24/7 (p < 0.01), while 24/7 did not differ from 24C (**Figures 10D–F**).

Both palisade (24C 7.1 ± 0.6, 24/7 13.3 ± 1.0, 24/13 21.6 ± 1.4) and spongy mesophyll (24C 6.0 ± 0.5, 24/7 11.4 ± 0.8, 24/13 16.2 ± 1.1) exhibited significant differences between all three lines (p < 0.01), confirming that both tissues follow a line-dependent gradient. In the midrib vascular bundle, JIM7 signal did not differ significantly between lines (p = 0.180), although qualitative observation suggested stronger labelling in 24/13 (**Figures 10D–F**).

In the petiole, all tissue types exhibited JIM7 epitopes (**Figures 10G–L**). Epidermal fluorescence showed an opposite trend compared with other tissues (24C 72.6 ± 3.0, 24/7 30.9 ± 1.5, 24/13 30.5 ± 1.7; p < 0.01), with the diploid control displaying significantly higher fluorescence than both tetraploid lines (p < 0.01), and 24/7 also exhibiting slightly higher values than 24/13 (p < 0.05). In collenchyma, 24/13 displayed the strongest labelling, significantly higher than 24C and 24/7 (p < 0.01). In petiole parenchyma, 24/13 was also significantly higher than both 24C and 24/7 (p < 0.01), with the difference between 24/13 and 24/7 being equally significant. In the petiole main vascular bundle, fluorescence differed significantly between lines (means ± SE: 24C 31.39 ± 0.74, 24/7 24.98 ± 0.24, 24/13 30.95 ± 0.85; p < 0.01). Tukey *post-hoc* showed that 24/7 was lower than both 24C and 24/13 (both p < 0.01), whereas 24/13 did not differ from 24C.

Taken together, both antibodies revealed clear line-dependent modulation of homogalacturonan epitopes, with JIM5 highlighting stronger differences in low-methyl-esterified pectin particularly in epidermal and vascular tissues (**Figure 9**), whereas JIM7 revealed pronounced accumulation of high-methyl-esterified pectin in mesophyll and petiole collenchyma/parenchyma, along with an unexpected reduction in the petiole epidermis of tetraploids compared with the diploid control (**Figure 11**).

**Figure 12:**
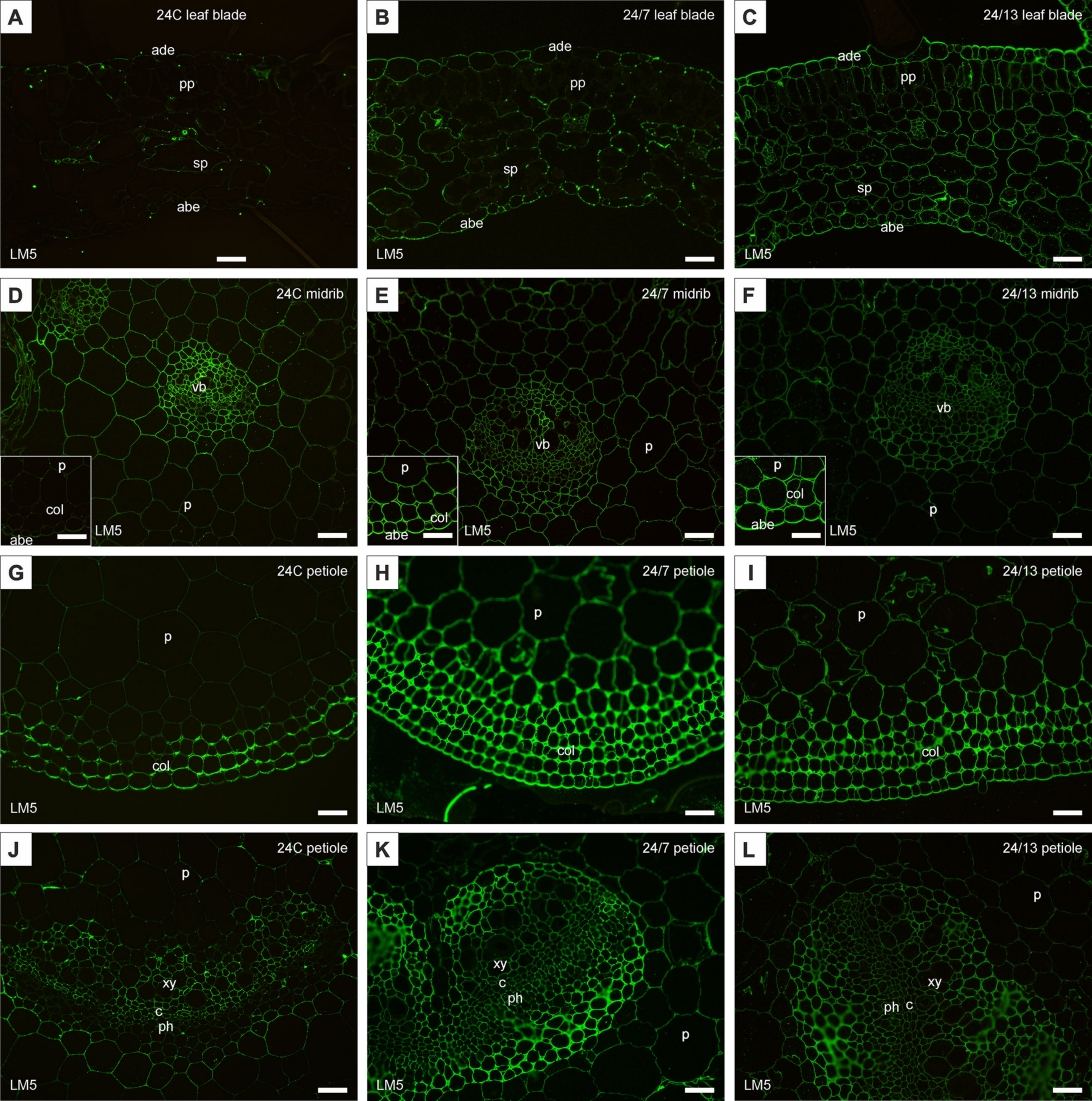
Immunohistochemical localization of LM5 epitope in control 24C **(A, D, G, J)** and tetraploid lines: 24/7 **(B, E, H, K)** and 24/13 **(C, F, I, L)** in the leaf lamina **(A–C)**, midrib **(D–F)** and petiole **(G–L)**; abe – abaxial epidermis, ade – adaxial epidermis, c – cambium, col – collenchyma, p – parenchyma, ph – phloem, pp – palisade parenchyma, sp – spongy parenchyma, vb – vascular bundle, xy – xylem. Scale bars **(A–L)**, insets **(D–F)** – 50 µm.

**Figure 13:**
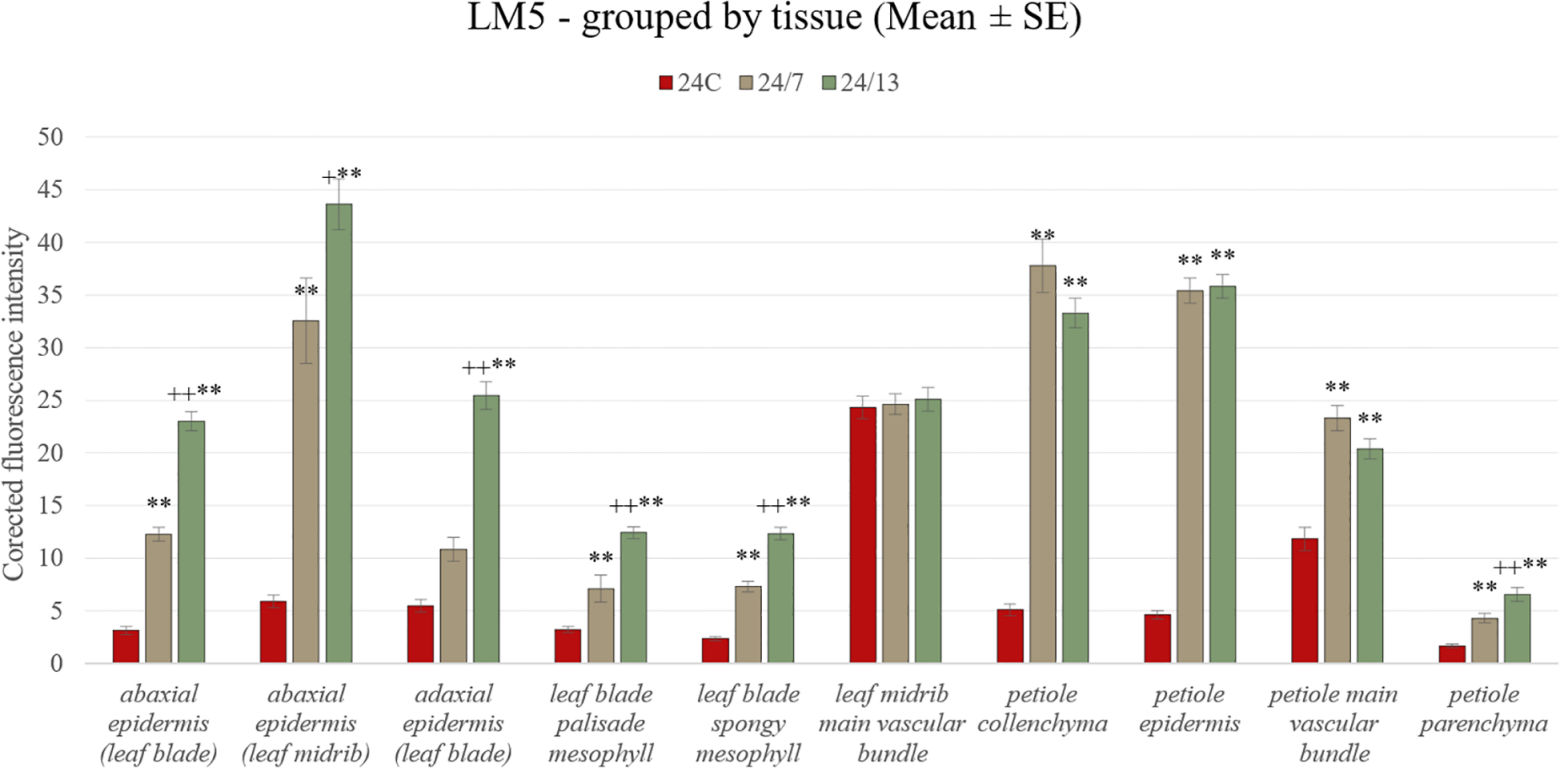
Quantification of LM5 fluorescence intensity in leaf and petiole tissues of diploid and tetraploid *Borago officinalis*. Corrected mean ± SE fluorescence intensities were determined for abaxial epidermis (leaf blade), abaxial epidermis (leaf midrib), adaxial epidermis (leaf blade), leaf blade palisade mesophyll, leaf blade spongy mesophyll, leaf midrib main vascular bundle, petiole collenchyma, petiole epidermis, petiole main vascular bundle, and petiole parenchyma. The LM5 antibody recognizes (1 → 4)-β-D-galactan side chains of rhamnogalacturonan I. Significant differences are indicated as (p ≤ 0.01 “**”) relative to the diploid control (24C), and (p ≤ 0.05 “+”; p ≤ 0.01 “++”) for comparisons between tetraploid lines (24/7 *vs* 24/13).

LM5 antibody, recognizing galactan side chains of RG-I, revealed clear line-dependent differences between diploid and tetraploid leaves (**Figures 12A–L**). In the abaxial epidermis of the leaf blade, fluorescence was markedly enhanced in tetraploids compared with the control (**Figure 13**) (mean ± SE: 24C 3.4 ± 0.5, 24/7 11.8 ± 1.6, 24/13 21.7 ± 1.1), with both 24/7 and 24/13 significantly exceeding 24C (p < 0.01), and 24/13 further surpassing 24/7 (p < 0.01). In the adaxial epidermis of the blade, a similar gradient was observed (24C 5.7 ± 0.5, 24/7 11.4 ± 0.9, 24/13 26.9 ± 1.0), with 24/13 significantly higher than both 24C and 24/7 (p < 0.01). In the abaxial epidermis of the midrib, 24/13 exhibited the strongest signal (44.4 ± 2.2) compared with 24C (6.2 ± 0.5) and 24/7 (33.2 ± 2.5), with both tetraploids exceeding the control, and 24/13 significantly higher than 24/7 (p < 0.01).

Within the palisade mesophyll, LM5 labelling showed a pronounced line-dependent increase (24C 3.2 ± 0.3, 24/7 6.7 ± 0.8, 24/13 12.0 ± 0.6), with both tetraploids significantly exceeding the control and 24/13 also surpassing 24/7 (p < 0.01). In the spongy mesophyll, fluorescence intensities followed the same pattern (24C 2.5 ± 0.2, 24/7 7.7 ± 0.6, 24/13 12.2 ± 0.5), again with both tetraploids significantly stronger than 24C and 24/13 higher than 24/7 (p < 0.01). In the main vascular bundle of the midrib, no significant differences between lines were detected (24C 24.5 ± 0.9, 24/7 24.6 ± 0.7, 24/13 25.6 ± 0.8).

In the petiole, LM5 epitopes were present in all tissues analyzed. In the epidermis, no significant differences were observed (24C 5.4 ± 0.4, 24/7 36.6 ± 1.3, 24/13 34.4 ± 1.1), although qualitative labelling appeared stronger in tetraploids (**Figures 9G–I**). In the collenchyma, differences were highly pronounced: 24/7 displayed the strongest fluorescence (38.5 ± 1.3), significantly higher than both 24C (4.7 ± 0.4) and 24/13 (30.9 ± 1.0; p < 0.01), while 24/13 also exceeded 24C (p < 0.01). In the parenchyma, a clear line-dependent increase was observed (24C 1.9 ± 0.2, 24/7 4.6 ± 0.4, 24/13 7.4 ± 0.5), with both tetraploids significantly higher than the diploid control (p < 0.01), and 24/13 also significantly higher than 24/7 (p < 0.01). In the main vascular bundle of the petiole, fluorescence intensities did not differ significantly (24C 14.0 ± 0.6, 24/7 23.3 ± 0.9, 24/13 20.6 ± 1.0).

Taken together, LM5 immunolabelling confirmed a pronounced increase in galactan epitopes across most tissues of tetraploid lines, with the most striking differences in both epidermal layers and mesophyll tissues of the leaf blade, as well as in the petiole collenchyma and parenchyma (**Figure 13**). These results indicate that polyploidization enhances RG-I galactan side chains, particularly in load-bearing and photosynthetic tissues, while vascular bundles remained comparatively unaffected.

The arabinan side chain of RG-I recognized by LM6 antibody, and the AGPs epitopes recognized by LM2 and JIM13 were not detected in any of the examined leaf samples.

## 4 Discussion

### 4.1 Scope and rationale of this study

Polyploidy can precipitate rapid, first-order shifts in cellular anatomy and primary cell-wall organisation that set the mechanical boundary conditions for growth before downstream metabolic reprogramming becomes detectable. Accordingly, this study concentrates on early consequences of genome doubling in *B. officinalis*: whole-leaf morphology and architectural traits, quantitative leaf anatomy including vascular attributes, and tissue-resolved immunolocalization of key wall epitopes—homogalacturonan methylesterification states (JIM5/JIM7), RG-I galactan side chains (LM5), and selected arabinogalactan proteins (LM2/JIM13)—which together influence extensibility, stiffness and growth anisotropy (Willats and Knox, 1999; Showalter, 2001; Wolf et al., 2009; Wormit and Usadel, 2018).

Our interpretation is anchored in quantified, statistically supported differences across lines. We draw on tissue-resolved fluorescence intensity measurements and on quantitative assessments of stomatal traits and trichome density to link organ-level form with tissue mechanics. This framework motivates a focus on early anatomical and cell-wall responses to genome doubling in *B. officinalis*.

### 4.2 Artificial polyploidization in medicinal plants: a general perspective

Polyploidization is a powerful and widely utilized tool in plant breeding, offering substantial advantages in terms of improving plant characteristics and increasing genetic diversity (Sattler et al., 2016; Trojak-Goluch et al., 2021). In particular, artificial genome doubling has been shown to induce notable changes in plant morphology and metabolic processes (Dhooghe et al., 2011; Tavan et al., 2015; Li et al., 2018; Homaidan Shmeit et al., 2020; Sabzehzari et al., 2020; Fernandes et al., 2023), which can be advantageous for medicinal plant production (Salma et al., 2017; Iannicelli et al., 2020; Madani et al., 2021). The manipulation of ploidy levels often results in the enlargement of plant organs (Levin, 2002), a characteristic that can be particularly beneficial for species of interest in pharmacological applications (Niazian, 2019), as it may lead to enhanced biomass production and improved yield of bioactive compounds (Lavania et al., 2012).

The doubling of the genome has also been associated with increased tolerance to both biotic and abiotic stresses, making polyploid plants more resilient in challenging environmental conditions (Tan et al., 2015). As with all other crops, breeding for resistance to both biotic and abiotic stresses is essential for medicinal plants (Lakshman Chandra, 2017). Furthermore, polyploid forms of medicinal plants expand the available germplasm base, offering breeders a broader genetic pool for selection and enhancement of desirable traits, including increased production of secondary metabolites that contribute to their therapeutic properties (Meru, 2012; Sattler et al., 2016).

As mentioned in the Introduction, *in vitro* methodologies for *B. officinalis* have been extensively studied (Knipp and Honermeier, 2002; Al-Mohammed Maher et al., 2014; Nasser et al., 2019; Quinn et al., n.d.), yet their reproducibility and efficiency remain variable. This variability may be partly attributed to the absence of certified cultivars or inbred lines in this species, as commonly available seed material is either open-pollinated or selected for ornamental traits rather than genetic uniformity. In our preliminary trials, attempts to establish cultures directly from nodal segments were largely unsuccessful, as extended sterilization led to tissue death while shorter sterilization increased the risk of contamination. Such observations underscored immature embryos as the most reliable explant source for initiating *in vitro* cultures of this species. In addition, explants cultured on MSvdSM medium (Van Der Salm et al., 1994) consistently performed better than those on standard MS, exhibiting fewer symptoms of chlorosis and necrosis. These results are consistent with previous reports demonstrating that FeEDDHA-based iron supplementation reduces chlorosis and improves chlorophyll content in various species (Christensen et al., 2008). Nevertheless, artificial polyploidization of this species remains largely unexplored. The application of genome doubling in medicinal plants has been shown to induce significant morphological and metabolic changes, often enhancing secondary metabolite production and stress resilience. These traits are particularly valuable for species cultivated for pharmacological purposes, as polyploid forms may exhibit improved biomass accumulation and altered bioactive compound profiles. The present study demonstrates that oryzalin-induced polyploidization in *B. officinalis* resulted in distinct phenotypic modifications, warranting further investigation into its potential implications for medicinal use and commercial cultivation.

### 4.3 Macroscopic morphological changes in polyploid sub-clones

Polyploidization is known to induce notable changes in plant morphology, often resulting in increased organ size, altered leaf shape, and modified plant architecture (Tang et al., 2010; Gupta et al., 2024; Neenu et al., 2024; Ræbild et al., 2024; Sanaei-Hoveida et al., 2024; Thriveni et al., 2024). In the present study, macroscopic traits were predominantly line-dependent: sub-clonal line 24/13 of *B. officinalis* displayed ovate leaves with more pronounced serration compared with both the diploid control and the other tetraploid line (**Figures 2**, **3**). Increased pubescence was confirmed quantitatively as higher trichome density in both tetraploids relative to the diploid control (Section 3.5).

Similar morphological effects have been reported in other artificially induced polyploids. For instance, colchicine-induced tetraploid *Stevia rebaudiana* exhibited more pubescent leaves with higher trichome density (Zhang et al., 2018). Likewise, polyploid lines of *Artemisia annua* (Lin et al., 2011), *Citrus junos* (Tan et al., 2015), *Citrus reticulata* (Tan et al., 2017) and *Echinacea purpurea* (Xu et al., 2014) developed different leaf morphology, while oryzalin-induced tetraploid *Rhododendron fortunei* displayed slower growth rates, thicker and rounder curled leaves (Mo et al., 2020). In our dataset, we did not detect a uniform increase in overall lamina thickness; rather, the epidermal layers (adaxial and abaxial) were significantly thicker in tetraploids, and the palisade parenchyma was markedly thicker in line 24/13 than in both 24C and 24/7, indicating a layer-specific anatomical response consistent with polyploid trends (Trojak-Goluch et al., 2021).

However, while polyploids often exhibit enlarged cells, this does not always result in an overall increase in plant size. Reduced cell division rates in polyploids can lead to more compact growth forms, as previously noted in various species (Horn, 2002; Sattler et al., 2016; Hias et al., 2017). A clear divergence in shoot architecture of the sub-clonal line 24/13 relative to both the diploid control (24C) and the tetraploid line 24/7 is evident (**Figure 3**). This observation is consistent with similar findings in other polyploid species, where genome doubling has been associated with reduced internode elongation and altered plant morphology. For instance, *Catharanthus roseus* polyploids exhibited a more compact growth pattern than their diploid counterparts (Xing et al., 2011). Likewise, triploid and tetraploid *Citrullus lanatus* plants displayed a more compact form compared to diploids, suggesting that genome duplication can lead to reduced internode elongation and altered plant architecture (Mahmud et al., 2024). This phenomenon has been reported across a range of polyploid species, including *Malus* (Hias et al., 2017), *Buddleja* (Rose et al., 2000), *Petunia* (Regalado et al., 2017), *Rosa* (Feng et al., 2017), *Platanus* (Liu et al., 2007), and *Eriobotrya* (Blasco et al., 2015), where polyploid individuals exhibited more compact growth habits compared to their diploid relatives. These modifications may offer practical advantages in controlled cultivation by allowing higher planting densities without compromising biomass production. However, further investigation is needed to determine how these morphological changes influence plant physiology, biomass accumulation, and secondary metabolite biosynthesis in *B. officinalis*. In addition to these macroscopic alterations, polyploidization also affects microscopic traits, particularly stomatal and trichome density, which are examined in the following sections.

### 4.4 Standard microscopic analyses: stomata, trichome and histological sections

#### 4.4.1 Analyses of stomata

Stomata play a crucial role in regulating gas exchange and water loss, and their density and size are frequently altered in polyploid plants (Moghbel et al., 2015; Salma et al., 2017; Švécarová et al., 2019; Iannicelli et al., 2020). Polyploidization often leads to larger but less densely distributed stomata, which may influence transpiration efficiency and drought tolerance (Byrne et al., 1981; Ahmadi and Ebrahimzadeh, 2020). In line with previous findings, polyploidization in *B. officinalis* resulted in significant modifications of stomatal traits. Tetraploid sub-clonal lines exhibited a notable increase in stomatal size, accompanied by a substantial reduction in stomatal density, a trend commonly observed in polyploid species. Increased stomatal size has been reported in *C. junos* and *C. reticulata* (Tan et al., 2015, 2017), *A. annua* (Lin et al., 2011), *C. roseus* (Xing et al., 2011), *Centella asiatica* (Kaensaksiri et al., 2011), and *Papaver somniferum* (Mishra et al., 2010). Similarly, *H. lupulus* (Švécarová et al., 2019) and *A. reptans* (Švécarová et al., 2018) exhibited significantly larger stomata in tetraploid and higher polyploid forms compared to diploid controls. The inverse relationship between stomatal size and density is a well-documented consequence of polyploidization and has been linked to altered leaf gas exchange dynamics and water use efficiency (Byrne et al., 1981).

Larger stomata may facilitate enhanced CO_2_ diffusion under favorable conditions, whereas lower stomatal density could contribute to reduced transpirational water loss, potentially improving drought resilience (Trojak-Goluch et al., 2021; Marks et al., 2024). In *B. officinalis*, it remains to be determined whether these changes confer an adaptive advantage under drought or other abiotic stress conditions. Further physiological studies, including assessments of gas exchange rates and water potential dynamics, will be essential to elucidate the ecological and agronomic significance of these stomatal modifications in tetraploid plants. In addition, the particularly pronounced stomatal morphology observed in line 24/13 may be linked to underlying differences in hormonal regulation (auxin–cytokinin balance) or epigenetic modifications, which warrants further investigation in future studies.

#### 4.4.2 Analyses of trichomes

Leaf trichomes contribute to mechanical and chemical defense, modify the leaf–air boundary layer and surface energy balance, and can mediate secretion/accumulation of specialized metabolites in glandular types, thereby influencing plant–environment interactions and stress responses (Wang et al., 2021).

Polyploidy is frequently associated with alterations in trichome traits, including increased density (Mtileni et al., 2021). In *B. officinalis*, abaxial trichome density was significantly higher in the tetraploid sub-clones than in the diploid control (24C 2.00 ± 0.09, 24/7 14.44 ± 0.51, 24/13 19.55 ± 0.63 trichomes mm^-^²; p ≤ 0.01; **Table 4**), indicating a line-dependent enhancement of indumentum development.

Comparable polyploidy-associated increases in trichome density have been documented across diverse taxa. In *Rhodohypoxis baurii*, polyploid individuals exhibited higher trichome density relative to diploids alongside larger and more numerous stomata, although water-use efficiency per se did not differ between ploidies under well-watered or deficit conditions (Mtileni et al., 2021). In *Thymus persicus*, *in vitro*-induced higher ploidy levels produced leaves with significantly increased trichome densities (and darker green color) (Tavan et al., 2015), consistent with earlier observations in induced tetraploids of *Tanacetum parthenium* (Majdi et al., 2010). Tetraploid *S. rebaudiana* similarly displayed higher glandular trichome density together with larger stomata and elevated chlorophyll indices (Zhang et al., 2018). In *Cannabis sativa*, tetraploids showed a ≈40% increase in trichome density on sugar leaves, accompanied by shifts in terpene profiles and a modest rise in CBD content (Parsons et al., 2019). Collectively, these precedents align with the present line-dependent increase in trichome density observed in *B. officinalis* and suggest that trichome traits may be a recurrent and tractable component of the polyploid phenotype.

Functionally, a denser indumentum can alter the leaf–air interface and surface microenvironment, with potential consequences for leaf energy balance, boundary-layer properties and mechanical protection (Karabourniotis et al., 2020). While such effects were not tested here, the anatomical baseline established in this study; including the layer-specific thickening of epidermal strata and the increased abaxial trichome density; provides a coherent framework for targeted follow-up, for example, by integrating micromorphology with optical, wetting and herbivory assays, and by assessing whether trichome-related surface traits interact with gas-exchange phenotypes documented in the stomatal analysis.

#### 4.4.3 Anatomical changes in tetraploids

In the presented studies, similarly to the other research on polyploids (Becker et al., 2022), polyploidization is frequently linked to the ‘gigas’ syndrome, namely enlarged cells and shifts in tissue proportions (Balao et al., 2011; Segraves, 2017). In our material, histology revealed a layer-specific reconfiguration of the lamina: the abaxial and adaxial epidermis were significantly thicker in tetraploids than in the diploid control, with a line-dependent pattern (24/7 > 24/13 > 24C); the palisade parenchyma was markedly thicker in 24/13 than in both 24C and 24/7, whereas 24/7 did not differ from 24C; the spongy parenchyma was thinner in 24/13 than in 24C and 24/7, with no difference between 24/7 and 24C, and without a uniform increase in total lamina thickness (**Figure 6**). These quantified patterns indicate a redistribution of lamina thickness towards the palisade domain in 24/13, a configuration that provides a concrete anatomical substrate for changes in leaf mechanics (greater resistance to strain at the surface; enhanced load-bearing in the upper mesophyll) without invoking whole-lamina enlargement (Porturas, 2018). Vascular traits showed a similar line-dependent scaling. The main midrib bundle area was larger in 24/13 than in 24C and also exceeded 24/7, whereas 24/7 did not differ from the control. Xylem vessel area increased in both tetraploids relative to 24C, with no difference between 24/7 and 24/13. In petioles, both total cross-sectional area and the central bundle area were higher in tetraploids than in 24C, with 24/13 > 24/7; vessel elements appeared wider and the cambial zone more conspicuous in tetraploids, particularly in 24/13. Such geometry plausibly increases theoretical hydraulic capacity and is consistent with wider vessels and inferred transport advantages reported in other polyploid systems (Barceló-Anguiano et al., 2021; Fonollá et al., 2023). The pattern also accords with hormone-linked modulation of cambial activity under polyploidy, including effects of gibberellins and cytokinins on cambial cell proliferation and vascular differentiation (Dudits et al., 2016).

Comparable reallocation of lamina tissues has been described elsewhere. In *Actinidia chinensis*, tetraploids showed epidermal enlargement together with *PME2* up-regulation that is consistent with shifts in homogalacturonan status, which resonates with our finding of thicker epidermal strata alongside the epitope repartitioning presented below (Zhu et al., 2024). In *Mangifera indica*, tetraploid leaves exhibited wider xylem vessels and altered mesophyll organisation, paralleling our increase in vessel area and the palisade-biased expansion in line 24/13 (Fonollá et al., 2023). Studies in *Oxalis* documented morpho-anatomical adjustments associated with polyploidy that provide a precedent for the tissue-level rebalancing recorded here, namely epidermal thickening, redistribution towards palisade and reduced spongy thickness in 24/13 (Castro et al., 2007; Krejčíková et al., 2013). More broadly, foundational syntheses emphasize that genome duplication can restructure plant form and function in ways that open new performance space, an interpretation consistent with the line-dependent anatomical trajectories observed in *B. officinalis* (Ramsey and Schemske, 1998; Levin, 2002; Ramsey, 2011).

Considered together, the quantified anatomical shifts in tetraploids of *B. officinalis* accord with broader patterns of structural and functional adjustment under polyploidization (Ramsey and Schemske, 1998; Becker et al., 2022). These data delineate tissue domains where changes in leaf mechanics and potential hydraulic capacity are most likely to arise, thereby advancing discussion from generalities to a tissue-resolved framework in this species (Tyree and Zimmermann, 2002; Onoda et al., 2011). While our study provides valuable insights into polyploidization, future investigations should also incorporate co-staining with DAPI to directly visualize ploidy levels and chromatin organisation *in situ*, as this would provide valuable complementary insights into the cellular consequences of polyploidization.

### 4.5 Immunolocalization of cell wall components

HG is the major pectin found in primary plant cell walls. Once incorporated into the cell wall matrix, HG undergoes further modifications that affect its properties (Wolf et al., 2009). One such modification is the demethylesterification of HG, cataly**z**ed by pectin methylesterases (PMEs), which remove methyl groups from the HG backbone. The reduction in cell wall stiffness observed in various plant organs is often associated with increased demethylesterification of pectins (Wormit and Usadel, 2018). In our study, HG epitopes were redistributed in a tissue- and line-dependent manner: JIM5 (low-methyl-esterified HG) increased across epidermal domains of the blade and midrib and in the palisade mesophyll, whereas differences were weak or absent in the spongy mesophyll and varied among petiole tissues. By contrast, JIM7 (high-methyl-esterified HG) showed line-dependent increases in the epidermis and mesophyll of the blade (strongest in 24/13), no change in the midrib bundle, and a reduction in the petiole epidermis in tetraploids relative to the diploid control. Taken together, these patterns indicate a repartitioning of HG methyl-esterification rather than a uniform shift with ploidy, consistent with the view that lower methyl-esterification stiffens walls via calcium cross-links, whereas higher methyl-esterification favors extensibility (Wolf et al., 2009; Peaucelle et al., 2011; Cosgrove, 2016; Wormit and Usadel, 2018). Accordingly, JIM5-enriched surfaces and vascular interfaces are consistent with local reinforcement at load-bearing boundaries, whereas JIM7-enriched mesophyll domains (particularly in line 24/13) are compatible with accommodation of the palisade expansion identified histologically. Polyploid-associated adjustment of HG signatures has also been reported in *Actinidia chinensis*, where *PME2* expression is up-regulated, providing a relevant precedent for the line- and tissue-specific HG changes we observe (Zhu et al., 2024). The LM5 epitope, associated with the galactan side chain in RG-I, may play a role in cell wall stiffening and stability, which is crucial for maintaining the structural integrity of plants and their ability to respond to mechanical stress (Willats and Knox, 1999). In our analysis, LM5 labelling was present in the diploid control but not uniformly across tissues, and it increased in the tetraploid lines in a tissue-resolved and line-dependent manner, with the strongest and most widespread signals in 24/13 (**Figure 13**). The most pronounced differences occurred in the epidermal layers and mesophyll of the blade, and in collenchyma and parenchyma of the petiole, whereas vascular bundles were comparatively less affected. Considered alongside the anatomically thicker epidermis and the palisade expansion in 24/13 (**Figure 6**), the LM5 gains indicate a coordinated reinforcement of matrix components in load-bearing and photosynthetic tissues that can maintain tissue integrity while accommodating dimensional scaling (Willats and Knox, 1999). The stronger LM5 signal in tetraploid leaves is consonant with observations in tetraploid *Mangifera indica*, where LM5 labelling has been reported and discussed in the context of water-stress adaptation (Fonollá et al., 2023). In *Arabidopsis thaliana*, comparative analyses across diploid, tetraploid and hexaploid lines indicate that increasing ploidy is associated with altered cell-wall composition, including higher pectin content, which provides a mechanistic backdrop for polyploid remodelling of matrix polysaccharides (Corneillie et al., 2019). A similar trend has been detected in autotetraploid *Oryza sativa*, where total pectin content was elevated relative to diploids, reinforcing the generality of ploidy-linked pectin reorganization in crops (Leng et al., 2023). Within this comparative context, the tissue-resolved LM5 increase documented here for *B. officinalis*, most pronounced in epidermal layers and mesophyll of the blade and in collenchyma and parenchyma of the petiole, supports a model in which RG-I galactan contributes to wall-matrix stabilization in load-bearing and photosynthetic tissues. Such a configuration is coherent with the anatomically thicker epidermis and the palisade expansion quantified in line 24/13, and it offers a plausible route by which genome duplication can rebalance stiffness and extensibility while preserving tissue integrity under variable mechanical or hydration conditions (Corneillie et al., 2019; Fonollá et al., 2023; Leng et al., 2023). In sum, by linking line-dependent, tissue-resolved changes in leaf anatomy with concordant shifts in pectic epitopes, this study moves the understanding of polyploid effects in *B. officinalis* from general description to a mechanistic framework that identifies where and how genome duplication can alter wall architecture, tissue mechanics and potential hydraulics without recapitulating broader, already established narratives.

## 5 Conclusion

This study demonstrates that oryzalin-induced tetraploidy in *B. officinalis* leads to pronounced anatomical and cellular changes affecting leaf morphology, stomatal traits, trichome density, vascular development, and cell wall composition. The observed enlargement of stomata, midrib and petiole vascular bundles, increases in xylem vessel area, reduction in stomatal density, and tissue-resolved remodelling of pectic epitopes (JIM5, JIM7) together with increased RG-I galactan (LM5) indicate that genome duplication restructures specific tissues that determine surface reinforcement, mesophyll extensibility, and potential hydraulic capacity. Among the tested lines, tetraploid sub-clone 24/13 exhibited the most pronounced alterations, including thicker adaxial and abaxial epidermis, palisade expansion, higher abaxial trichome density, and enlarged midrib and petiole bundle areas with greater xylem vessel area. Taken together, these line-dependent, tissue-resolved shifts provide a quantitative, mechanistic framework linking wall chemistry to anatomy in this species and identify concrete targets for follow-up tests of mechanics and hydraulics. The results presented here contribute to a better understanding of polyploidy-induced anatomical adaptation and offer a valuable foundation for further biotechnological applications and physiological studies in *B. officinalis* and other medicinal plants.

## Data Availability

The raw data supporting the conclusions of this article will be made available by the authors, without undue reservation.
